# Real-Time Embedded Smart-Particle Monitoring for Index-Based Evaluation of Asphalt Mixture Compaction Quality

**DOI:** 10.3390/s26061822

**Published:** 2026-03-13

**Authors:** Min Xiao, Xilan Yu, Wei Min, Fengteng Liu, Yongwei Li, Haojie Duan, Feng Liu, Hairui Wu, Xunhao Ding

**Affiliations:** 1 Jiangxi Communications Investment Group Co., Ltd., No. 367, Chaoyangzhou Middle Road, Nanchang 330025, China; xiaom123555@163.com (M.X.); yuxl163@163.com (X.Y.); weim880905@163.com (W.M.); liyw123876@163.com (Y.L.); 2 School of Transportation, Southeast University, Nanjing 211189, China; 220233397@seu.edu.cn (F.L.); 220245388@seu.edu.cn (H.D.); 220253449@seu.edu.cn (F.L.); whr2214@my.swjtu.edu.cn (H.W.)

**Keywords:** embedded smart particle, asphalt mixture, vibratory compaction, real-time monitoring, orientation sensing

## Abstract

**Highlights:**

**What are the main findings?**

- The embedded smart particle remains stable at 165 °C and effectively quantifies the “rapid-to-slow” densification phases via attitude-angle evolution.
- Nonlinear machine learning models using orientation features successfully reconstructed the real-time compaction degree with high accuracy (R^2^ > 0.960).

**What are the implications of the main findings?**

- This method enables continuous in situ monitoring of pavement quality, solving the time lag problem of traditional post-compaction testing.
- The proposed sensing framework provides a reliable data foundation for real-time feedback control in intelligent transportation infrastructure construction.

**Abstract:**

Compaction quality governs asphalt pavement durability, but conventional density checks are intermittent. Reliable compaction control of asphalt mixtures requires real-time information on internal responses rather than relying solely on endpoint density measurements. In this study, an embedded smart-particle framework is developed for in situ monitoring and index-based evaluation of vibratory compaction quality, integrating multi-source sensing, feature extraction, and compaction degree mapping. The smart particle integrates inertial/orientation sensing together with thermal–mechanical measurements, and its high-temperature survivability and calibratability are verified through thermal exposure and calibration tests. During laboratory vibratory compaction of representative asphalt mixtures, raw signals are converted into stable attitude responses via attitude estimation and filtering; posture-dominant descriptors are then extracted and used to establish a data-driven mapping from internal responses to compaction degree using regression models. Results show that the device remains stable under typical hot-mix asphalt conditions, with calibration exhibiting high linearity (temperature channel R^2^ > 0.990; force channel R^2^ > 0.980 in the relevant range). Filtering markedly enhances inertial-signal usability under strong vibration and improves the interpretability of attitude-response evolution during compaction. The evolution of attitude features is consistent with the “rapid-to-slow densification” process, yielding correlations of |r| ≈ 0.35–0.47 with compaction degree evolution. Nonlinear regressors outperform linear baselines, and the better-performing nonlinear models achieve strong predictive performance across all six specimens, with R^2^ values reaching 0.740–0.960 and RMSE reaching 0.016–0.043. Moreover, machine-learning-based feature-importance analysis reveals distinct mixture-type-dependent characteristics, indicating that AC and SMA transmit compaction-state information through partly different dominant response features. These findings demonstrate the feasibility of embedded smart particles for online compaction-quality evaluation and provide a basis for real-time feedback in intelligent compaction.

## 1. Introduction

The construction quality of asphalt pavements directly affects their service life. Compaction quality is one of the most important factors. Good compaction improves stiffness, strength, and stability. Poor compaction often causes rutting, cracking, and raveling. Recent studies show that reliable compaction indicators reflect particle rearrangement inside the mixture and relate closely to pavement performance [1].

Researchers first focused on understanding the internal densification process. The discrete element method (DEM) has become a useful tool for this purpose. Zhou et al. [2] used accelerated DEM models to study the consistency of internal structures during compaction. Sha et al. [3] linked densification behavior with particle dynamic responses during gyratory compaction. Experimental work combined gyratory compaction with X-Ray CT. These studies showed that different gradations follow different densification paths [4]. Dai et al. [5] further analyzed void characteristics during compaction and described a progressive reduction in air voids within the mixture. Sun et al. [6] tracked particle motion and reported gradual structural evolution. Liu et al. [7] used CT imaging and confirmed that the gradual change in air voids reflects internal stability during deformation. Wang et al. [8] also reported a three-stage evolution of air voids under high-temperature loading.

Field construction monitoring has also developed quickly. Roller-mounted ground-penetrating radar systems can measure density in real time and provide continuous spatial coverage [9]. Multi-domain intelligent compaction measurements have been used to improve cross-layer quality evaluation [10]. These techniques help bridge laboratory observations and field practice.

From a micromechanical view, load transfer depends on aggregate contacts. Wan et al. [11] proposed a three-dimensional contact quantification method based on CT images. Zheng et al. [12] introduced topological analysis to describe aggregate contact networks more rigorously. Mixture workability and particle shape also influence compaction. Chen et al. [13] showed that workability affects aggregate distribution and asphalt film uniformity. Ding et al. [14] reported that coarse aggregate morphology changes mixture flowability and compaction efficiency.

DEM simulations provide more details at the particle scale. Al Khateeb et al. [15] simulated porous asphalt compaction and observed a shift from sliding to interlocking behavior. Zhang et al. [16] measured aggregate acceleration responses and identified compaction mechanisms. Su et al. [17] studied aggregate migration and self-organization and showed that collective motion controls densification. Xue et al. [18] reviewed current DEM modeling strategies and summarized contact models and applications. Xie et al. [19] used DEM to analyze segregation and nonuniformity in mixtures.

Particle forces and particle shapes also play key roles. Wang et al. [20] investigated force transmission during compaction and showed that elongated and flat particles change stress paths. Wan et al. [21] developed algorithms to generate realistic virtual asphalt mixtures for numerical modeling. Chen et al. [22] experimentally studied coarse-grained rearrangement during vibratory compaction and observed staged structural reorganization.

Despite these advances, two limitations remain. Most methods rely on CT imaging, post-test analysis, or offline simulations. These approaches are difficult to use in real time on construction sites. Many indicators also focus on only one physical quantity, such as density or stiffness. They cannot capture structural, mechanical, and thermal states at the same time.

This study aims to fill these gaps by designing an Smart particle sensor with integrated capabilities. It can be used to monitor 3D attitudes, triaxial forces, and temperature during vibratory compaction. It can also apply filtering methods, feature extraction, and regression analysis to establish correlations between multi-source information and real-time compaction degree. It can be used as a tool for intelligent construction and quality control.

## 2. Experiment Preparation

### 2.1. Development and Verification of the Integrated Sensor

The device was built around an inertial measurement unit (IMU) and a thin-film piezoresistor with its control circuit. Modules for triaxial acceleration, geomagnetic sensing, temperature, and three-directional pressure measurements were integrated. The sensor adopted a cubic double-layer frame package with a side length of 2.8 cm to ensure stability within the specimen. To withstand the high-pressure and high-temperature environment during asphalt mixture compaction, the outer package frame was made of glass-fiber-reinforced nylon, while the inner frame was made of aluminum alloy. The glass-fiber-reinforced nylon was selected because it maintained strength at elevated temperatures and allowed slight deflection under pressure, while the aluminum alloy inner frame provided high rigidity, ensuring that the piezoresistor maintained a proper stress response. The results of the material tests will be included in the Supplementary Information. Prior feasibility tests demonstrated that five fabricated particles survived embedding in hot-mix asphalt and repeated vibratory compaction, with each particle successfully enduring more than four repeated trials, confirming the mechanical integrity and functional stability of the chosen materials.

**Figure 1:**
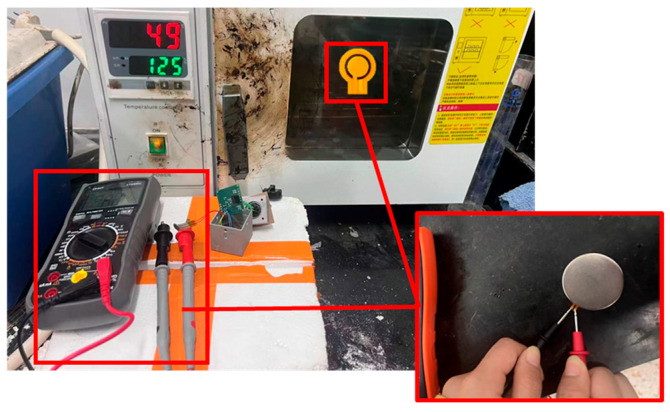
High-temperature performance test of components.

The formation mechanism and monitoring feasibility of aggregate skeleton structures in asphalt mixtures have been systematically investigated in previous work. Based on sphere-packing theory, a quantitative framework using a weighted-average particle-size index was established to evaluate skeleton stability across gradations. The evolution of coordination number, force-chain distribution, and fabric tensor was analyzed by penetration tests and DEM simulations of skeleton-controlled mixtures, indicating that an appropriate gradation ratio can enhance both longitudinal bearing capacity and skeleton stability. Furthermore, a skeleton–sensor coupling model verified numerically that an embedded particle can effectively capture sliding, rotation, and rearrangement behaviors of aggregates, providing a solid theoretical foundation for the subsequent experimental investigations presented here. All internal working components were made of heat-resistant materials, and the overall performance has been validated through a series of high-temperature tests, which specifically include the following:

(1) High-temperature performance test of components: First, apply different pressures to the piezoresistor at ambient temperature and record the resistance values; then, place it in a constant-temperature oven and maintain it at 100 °C/125 °C for 1 h; after cooling to ambient temperature, test the resistance change again to evaluate its thermal stability. The experimental procedure is illustrated in Figure 1.
(2) High-temperature performance test of the smart particle: The fully assembled intelligent particle was embedded in 165 °C asphalt mixture and was subsequently retrieved after the specimen naturally cooled to ambient temperature. Three-sided pressing tests were conducted both before and after the heating–cooling process to verify the consistency and validity of its response.

**Figure 2:**
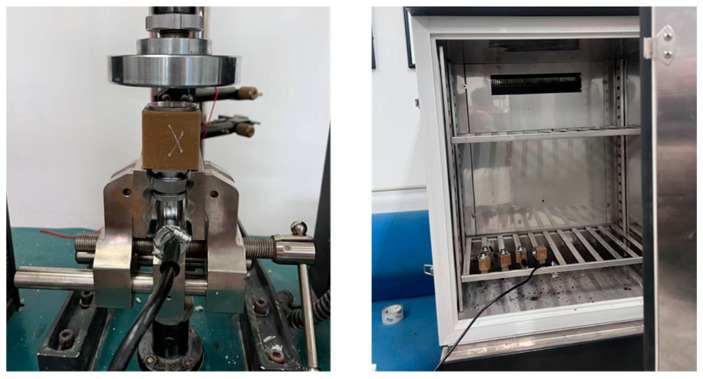
Temperature and pressure calibration.

Through the attitude solution algorithm, the sensor output real-time pitch, roll, and yaw angle (pitch (θ), roll (ϕ), and yaw (ψ) denote the Euler angles representing rotation about the sensor’s x-, y-, and z-axes, respectively, under the right-handed sensor coordinate system), while the voltage output of the thin-film piezoresistor was used to determine the loading trend of the smart particle, thereby providing a data basis for the dynamic identification of asphalt mixture compaction. To ensure the accuracy of monitoring information, the smart particle was subjected to temperature and pressure calibration to verify the validity of its monitoring data:

(1) Mechanical calibration: Using an electronic universal testing machine, the smart particle is continuously loaded within the range of 0–500 N, with the press pressure time-series data exported. Simultaneously, the smart particle continuously collects the piezoresistor output voltage time-series data, and the two sets of data are matched in the time domain to complete the calibration of the mechanical module.
(2) Temperature calibration: The smart particle was kept in a constant-temperature environmental chamber, and the voltage signal of its temperature-sensitive resistor was tested at different temperatures to calibrate the temperature monitoring module of the smart particle. Two types of calibration devices are shown in Figure 2.

Subsequently, the external transmission of the smart particle was designed with an external cable diameter of 7 mm, utilizing an RS-485 bus for signal output to enhance anti-interference capability and data transmission stability. The entire device was managed by the host computer, which performed unified data acquisition and storage.

### 2.2. Material Preparation

According to the Technical Specifications for Construction of Highway Asphalt Pavements (JTG F40-2004) [23], three types of asphalt mixtures were prepared in this study, namely AC-13, AC-16, and SMA-16. The raw materials consisted of basalt aggregate and #90 petroleum bitumen. Specifically, one specimen was prepared for AC-13, three specimens were prepared for AC-16, and two specimens were prepared for SMA-16, giving a total of six specimens. The mixing temperature was 165 °C.

The basic properties of the aggregates and bitumen are presented in Table 1. The aggregate used in this study was basalt, which exhibited low crushing and abrasion values and thus provided adequate strength and durability for the asphalt mixtures. The binder was #90 petroleum bitumen, whose penetration, ductility, and softening point all satisfied the specification requirements. The gradation schemes are listed in Table 2. The asphalt–aggregate ratios were 0.045 for AC-13, 0.050 for AC-16, and 0.048 for SMA-16.

## 3. Experiment and Methodology

### 3.1. Dynamic Monitoring of Sensor Signals and Mixture Compaction Degree Based on Vibratory Compaction Testing

**Figure 3:**
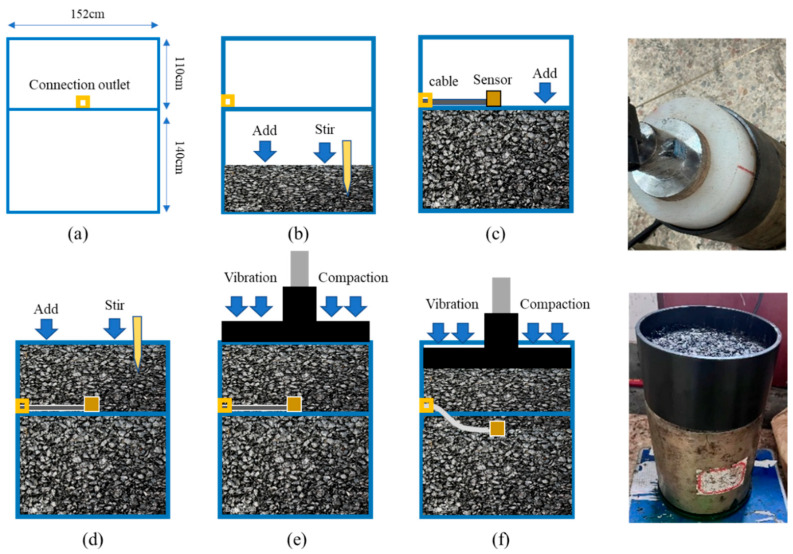
Smart-particle embedding and mixture compaction process: (**a**) The position of the hole; (**b**) rodding; (**c**) placed the smart particle and run the wire; (**d**) filling with asphalt mixture; (**e**,**f**) vibratory compaction and height recording.

To investigate the internal response and compaction evolution of asphalt mixture under vibratory compaction, smart particles were embedded in specially designed test mold buckets for in situ monitoring, and standard Marshall specimens were prepared for compaction degree calculation. Throughout the vibratory compaction process, the smart particle continuously outputs time-series data of attitude angles, temperature, and three-directional pressures. The preparation of Marshall specimens and the vibratory compaction process followed the Test Methods of Bitumen and Bituminous Mixtures for Highway Engineering (JTG E20-2011) [24], and the detailed testing procedure is as follows:

(1) Standard Marshall specimens were prepared using AC and SMA mixtures with standard Marshall compaction (mixing temperature 160 °C; compaction temperature 150 °C; 75 blows per side), and the density of the standard Marshall specimens ($\rho_{0}$) was measured as the denominator for the calculation of compaction degree.
(2) Six batches of asphalt mixture were prepared for vibratory compaction testing, corresponding to specimens AC-13, AC-16①, AC-16②, AC-16③, SMA-16①, and SMA-16②. The mass of each batch was measured before testing, and the mold temperature was maintained above 150 °C to minimize temperature loss during specimen preparation and loading.
(3) The specially designed test mold bucket was taller than a standard large Marshall mold, with an opening between the double-layer molds to guide out the sensor leads. The asphalt mixture was placed into the bucket in batches and tamped. The smart particle was embedded in the mixture with its top surface located approximately 4–6 cm below the surface. The embedding procedure and vibratory compaction process are illustrated in Figure 3.
(4) Vibratory compaction was performed using a model-1988, 15.5 kg electric tamping hammer (vibration frequency: 60 Hz; loading duration: >1 min). The equivalent height change in the specimen was synchronously recorded using a high-modulus nylon cylindrical gauge block (with a diameter equal to the mold’s inner diameter) and a high-definition camera, thereby obtaining the specimen’s volume during compaction and approximately calculating its real-time density.

The final dataset obtained from the compaction process included the following:

Density–time curve; attitude-angle sequence $\Theta \left(t\right)=\left[pitch,\;roll,\;yaw\right]$; three-directional pressure sequence $p\left(t\right)=\left[p_{x},\;p_{y},\;p_{z}\right]$.

Obtain the compaction degree–time curve based on the calculation formula for asphalt mixture compaction degree K(t):

(1) $$K\left(t\right)=\frac{\rho \left(t\right)}{\rho_{0}}$$

### 3.2. Data Conversion and Filtering

#### 3.2.1. Estimation of Three-Axis Attitude Angles

The raw data output by the smart particle sensor comprise triaxial acceleration and triaxial geomagnetic signals, which do not directly represent the sensor’s spatial attitude. To obtain the pitch, roll, and yaw angles, the raw data must be processed using an attitude determination algorithm.

First, the triaxial components from the accelerometer and magnetometer are normalized to eliminate dimensional effects and enhance comparability across measurement channels:

(2) $$a^{b}=\left[\begin{array}{c} a_{x}\\ a_{y}\\ a_{z} \end{array}\right],m^{b}=\left[\begin{array}{c} m_{x}\\ m_{y}\\ m_{z} \end{array}\right]$$

(3) $${\hat{a}}^{b}=\frac{a^{b}}{\left\|a^{b}\right\|},{\hat{m}}^{b}=\frac{m^{b}}{\left\|m^{b}\right\|}$$

On this basis:

(1) Estimation of pitch and roll: Based on the relationship between the gravity direction and the sensor coordinate frame, the triaxial accelerometer components can be used to compute roll and pitch. This step ensures that under static or low-dynamic conditions the attitude angles accurately reflect the sensor’s inclination.
(2) Estimation of yaw: After obtaining pitch and roll, their coupling effects must be further removed, and the triaxial magnetometer data are then employed to determine yaw. This procedure effectively avoids rotation-induced biases, enabling the yaw angle to correctly represent the sensor’s azimuth relative to geomagnetic north.
(3) Magnetic declination correction: Because the geomagnetic field exhibits declination differences across regions, the final yaw result must be corrected using the experimental area’s magnetic declination to ensure geographic consistency of the computations. Compute the roll angle ϕ and pitch angle θ from the gravity direction; after removing the effects of pitch and roll, compute the yaw angle ψ from the geomagnetic direction.

(4) $$\phi =arctan\left(a_{y},a_{z}\right)$$

(5) $$\theta =arctan\left(-a_{x},\sqrt{a_{y}^{2}+a_{z}^{2}}\right)$$

(6) $$\begin{array}{c} m_{x}^{c}=m_{x}\cos\theta +m_{z}\sin\theta \\ m_{y}^{c}=m_{x}\sin\phi \sin\theta +m_{y}\cos\phi -m_{z}\sin\phi \cos\theta \end{array}$$

(7) $$\psi =arctan\left(-m_{y}^{c},m_{x}^{c}\right)+\delta_{D}$$

Here, $\delta_{D}$ denotes the magnetic declination.

Through the above steps, the raw acceleration and magnetic data can be reliably transformed into a three-dimensional attitude-angle sequence, providing foundational data support for subsequent signal filtering, feature extraction, and compaction degree mapping.

#### 3.2.2. Signal Filtering Methods

During the vibratory compaction of asphalt mixtures, the Euler-angle signals recorded by the smart particle comprise two components: (i) a slowly varying trend caused by the mixture’s progressive densification, which reflects the overall evolution of the compaction process; (ii) pulse-like disturbances generated by the instantaneous impacts of the vibratory rammer, superimposed with environmental noise arising from the ambient magnetic field. If the raw signals are analyzed directly, transient disturbances often obscure the true trend. Therefore, the signals must be preprocessed to separate the effective trend and suppress transient noise. The specific steps are as follows:

(a) Preprocessing of raw data.

Transient disturbance detection: First, apply the stationary wavelet transform (SWT) to the raw accelerometer and magnetometer data to unfold signal features across multiple scales. Considering that the primary energy of the vibratory rammer impacts lies in the 2–30 Hz band, select the corresponding sets of scale coefficients to compute the energy envelope:

(8) $$E\left(t\right)=\sqrt{\sum_{j\in J}{\left(d_{j}^{+}\left(t\right)\right)}^{2}}+\delta$$

Here, $d_{j}^{+}\left(t\right)$ denotes the positive half-wave coefficient at scale j, and δ is a small offset used to suppress minor jitter.

Subsequently, the median absolute deviation (MAD) was used for threshold estimation, and morphological closing was applied to smooth the energy envelope, thereby identifying the intervals of transient disturbances.

At a sampling rate of $f_{s}=100Hz$, apply the stationary wavelet transform (SWT or MODWT) to the observed signal Θ(t) to obtain the detail coefficients $w_{j}[n]$ at each level. Based on the analysis of actual compaction curves, the duration of transient disturbances $T_{p}$ is approximately 30–500 ms, with the main energy concentrated in the 2–30 Hz band; the corresponding set of DWT scale coefficients is as follows.

(9) $$J=\left\{j_{L},\ldots ,j_{U}\right\},\;j_{L}=2,\;j_{U}=5$$

Apply positive half-wave rectification to these scales and construct the energy envelope.

(10) $$w_{j}^{+}\left[n\right]=\max\left(w_{j}\left[n\right]-\kappa_{j},0\right),E\left[n\right]=\sqrt{\sum_{j\in J}{\left(w_{j}^{+}\left[n\right]\right)}^{2}}$$

Here, $\kappa_{j}=0\sim 0.1\sigma$ is a small bias coefficient used to suppress minor positive-going jitter. Estimate the envelope noise and threshold using the median-based MAD:

(11) $${\hat{\sigma }}_{E}=\frac{Median\left(\left|E-Median\left(E\right)\right|\right)}{0.6745},\tau =c_{det}{\hat{\sigma }}_{E}\sqrt{2\ln N}$$

Here, $c_{det}$ is an adjustable threshold parameter. To mitigate the influence of small spikes, apply morphological closing to smooth the signal and identify transient time intervals that more closely reflect actual conditions.

(12) $$I^{c}=Close\left(I,L_{s}\right),while\;I=\left\{n\;\right|\;E\left[n\right]>\tau \}$$

Here, the structuring element is $L_{s}=T_{p}f_{s}$; based on the foregoing empirical parameter analysis, $L_{s}$ is approximately 3–50 samples (points).

Transient disturbance suppression: After the transient intervals are identified, the converted attitude-angle signals are further processed by high-amplitude compression and smoothing filtering so as to emphasize the slowly varying compaction-related trend while preserving the main evolution trajectory of the particle posture. In the present study, this trend-preserving route was taken as the primary preprocessing scheme for subsequent feature extraction. Asymmetric Wavelet Thresholding (AWT) was additionally considered as an auxiliary enhancement method for suppressing residual spikes when necessary, whereas Transient Masking and Interpolation (TMI) was examined mainly for comparison because excessive masking may over-smooth short-term structural rearrangement responses that are physically meaningful during compaction.

1. 1. Asymmetric Wavelet Thresholding (AWT)

Apply one-sided thresholding to coefficients at different decomposition levels to attenuate impact pulses while preserving the overall trend. This method effectively suppresses local impacts while retaining low-frequency components:

(13) $${\tilde{d}}_{j}\left(t\right)=\left\{\begin{array}{c} \mathrm{sgn}\left(d_{j}\left(t\right)\right)\cdot \max\left(\left|d_{j}\left(t\right)\right|-\lambda_{j}^{+},0\right),\;\;d_{j}\left(t\right)>0\\ \mathrm{sgn}\left(d_{j}\left(t\right)\right)\cdot \max\left(\left|d_{j}\left(t\right)\right|-\lambda_{j}^{-},0\right),\;\;d_{j}\left(t\right)<0 \end{array}\right.$$

Here, $\lambda_{j}^{+}$ and $\lambda_{j}^{-}$ are the thresholds in the positive and negative directions, respectively. When the impact is dominated by positive-going pulses, one may set $\lambda_{j}^{+}>\lambda_{j}^{-}$ to more strongly suppress positive half-wave transients, and vice versa. The reconstructed signal is

(14) $$\hat{\Theta }\left(t\right)=a_{J}\left(t\right)+\sum_{j=1}^{J}{\tilde{d}}_{j}\left(t\right)$$

2. Transient Masking and Interpolation (TMI)

Directly mask the transient intervals and reconstruct the signal using weighted smoothing spline interpolation. Control the interpolation outcome via a second-order difference operator and an adjustable smoothing parameter, thereby obtaining a signal that better follows the true trend; the corresponding formula is

(15) $$\hat{\Theta }\left(t\right)=\underset{z}{min}\;\sum_{n}\;w_{n}{\left(x[n]-z[n]\right)}^{2}+\alpha \sum_{n}\;{\left(\Delta^{2}z\left[n\right]\right)}^{2}$$

Here, $\Delta^{2}$ denotes the second-order difference operator, and α > 0 is the smoothing parameter. Let $x_{fill}\left[n\right]$ denote the signal after infilling with z[n].

Apply level-dependent MAD and two-sided soft-thresholding to $x_{fill}[n]$ (consistent with the AWT step), and reconstruct to obtain $\hat{s}[n]$.

To evaluate the filtering performance, RMSE and smoothness were used for a comprehensive analysis. RMSE gauges how close the filtered result is to the raw signal, while smoothness reflects whether high-frequency components have been effectively removed. Since the goal of filtering is to recover, as far as possible, the overall slowly varying trend within the subgrade during compaction, the ideal result should keep the smoothness as small as possible while ensuring that the RMSE relative to the raw signal does not become excessively large. The computation of these two metrics is as follows:

(16) $$RMSE=\sqrt{\frac{1}{N}\sum_{i=1}^{N}{\left({\hat{x}}_{i}-x_{i}\right)}^{2}}$$

(17) $$Smoothness=Var\left({\left\{{\hat{x}}_{i+1}-{\hat{x}}_{i}\right\}}_{i=1}^{N-1}\right)$$

Meanwhile, compute the difference in smoothness between the filtered signal and the raw signal to obtain; the larger the absolute value, the better the filtering effect.

(b) Next, apply high-amplitude compression and smoothing filtering to the converted attitude data to emphasize the slowly varying attitude signal.

1. Signal description

Assume that the observed sequence of pose changes due to compaction is x[n], which can be decomposed into three components:

(18) $$x\left[n\right]=s\left[n\right]+v\left[n\right]+o\left[n\right]$$

Here, s[n] denotes the true trend formed by gradual accumulation during compaction (a low-frequency/stationary component), which is the focus of this study. v[n] represents approximately zero-mean high-frequency environmental noise, whereas o[n] denotes sparse transient disturbances induced by the vibratory rammer and characterized by a non-zero mean. The goal of the data preprocessing is to compress large-amplitude outliers and suppress high-frequency noise so that the signal converges around the “baseline,” thereby highlighting the overall trajectory of s[n].

2. Baseline and Scale Estimation

Compared with the mean/standard deviation, the median and MAD (median absolute deviation) are insensitive to outliers and are suitable as robust estimators of the “baseline” and “scale”.

Baseline (location): $m=median\left\{x\left[n\right]\right\}.$

Scale (dispersion): $MAD=median\left\{\left|x\left[n\right]-m\right|\right\}.$

The complete parameter settings used in the signal filtering and attitude-angle smoothing procedures are summarized in the Attachment.

### 3.3. Feature Setup

After completing the filtering, the attitude and pressure signals can stably reflect the overall trend of the compaction process. Prior feasibility tests systematically evaluated the thermal performance of the smart-particle, showing that during vibratory compaction in asphalt mixture, the internal working modules did not exceed 80 °C, and signal outputs were effectively unaffected by temperature drift within this range. Accordingly, the compaction index input is derived solely from posture-related features, while stress is employed for the qualitative interpretation of force-chain reorganization, and temperature is monitored to assess the thermal condition of the device and ensure reliable operation. To further reveal the dynamics of compaction and establish their relationship with the compaction degree, this study constructs a feature set from the attitude and pressure signals:

Attitude features A: Includes cumulative angle drift (Δpitch, Δroll, Δyaw), angular velocity and angular acceleration (i.e., first- and second-order differences), moving-window root-mean-square (RMS) and standard deviation, local variance, composite tilt (e.g., $\sqrt{\left({pitch}^{2}+{yaw}^{2}\right)}$), and short-time energy and band-energy indices (spectral energy distribution, 2–30 Hz).

Pressure features P: Characterized mainly by the random occurrence of peaks, which shows limited correlation with the compaction process; therefore, only qualitative analysis was conducted in this study.

After filtering, the attitude-angle signals could more stably reflect the overall trend of the compaction process. However, to link these raw angular data to the compaction degree, it was still necessary to extract a set of indicators that characterized the dynamics of compaction. Based on the three-dimensional attitude angles, pitch (θ), roll (ϕ), and yaw (ψ), multiple feature quantities were designed and computed. These features not only capture the temporal evolution of the attitude signals but also possess clear engineering and physical meaning. The following features are selected for subsequent feature analysis and model construction:

(1) Original features:

(19) $$\theta \left(t\right),\phi \left(t\right),\psi \left(t\right)$$

(2) Variation relative to the initial angles: This feature captures the overall offset of the sensor attitude over the compaction cycle and can reveal whether the specimen exhibits a sustained settlement or tilting trend under compaction.

(20) $$\Delta \theta =\theta \left(t\right)-\theta \left(t_{0}\right),\;\Delta \phi \left(t\right)=\phi \left(t\right)-\phi \left(t_{0}\right),\;\Delta \psi =\psi \left(t\right)-\psi \left(t_{0}\right)$$

(3) Furthermore, by applying first-order differencing to the attitude angles, angular-velocity features can be obtained (the same applies to ϕ and ψ). Angular velocity reflects the rate and intensity of instantaneous responses during compaction; abrupt changes are often associated with vibratory impacts or sudden positional shifts in the asphalt mixture at the smart particle’s surface.

(21) $$\dot{\theta }\left(t\right)\approx \frac{\theta \left(t\right)-\theta \left(t-\Delta t\right)}{\Delta t}$$

(4) Based on this, a composite angular-velocity magnitude can be further constructed to provide a measure of the overall rate of attitude change:

(22) $$\Omega \left(t\right)=\sqrt{\theta {\left(t\right)}^{2}+\phi {\left(t\right)}^{2}+\psi {\left(t\right)}^{2}}$$

(5) To characterize short-term variations in attitude intensity, a windowed root-mean-square (RMS) feature is introduced to quantify the activity of attitude changes within a short-time window of length N (similarly for ϕ and ψ). This feature can be interpreted as a local energy metric, used to gauge the intensity of asphalt mixture compaction over a given time interval.

(23) $$RMS_{\theta }\left(t\right)=\sqrt{\frac{1}{N}\sum_{k=t-\frac{N}{2}}^{t+\frac{N}{2}}\dot{\theta }{\left(k\right)}^{2}}$$

(6) Beyond dynamic variations, the combinational relationships among attitude angles can also reflect compaction uniformity; therefore, a biaxial local tilt index is defined. Taking the composite of the pitch and yaw angles as an example, this index characterizes the magnitude of the sensor’s offset in the pitch–yaw plane; a larger value indicates stronger lateral displacement of the material in that region.

(24) $$T\left(t\right)=\sqrt{\theta {\left(t\right)}^{2}+\psi {\left(t\right)}^{2}}$$

Composites of the roll–pitch and roll–yaw angles were also included to ensure analytical comprehensiveness.

(7) The local angle variance based on a sliding window quantifies the short-term variability of the attitude angles within the window (similarly for ϕ and ψ). A larger variance indicates pronounced undulations in the attitude trajectories, which may be associated with local heterogeneities in the material structure.

(25) $$Var_{\theta }\left(t\right)=\frac{1}{N}\sum_{k=t-\frac{N}{2}}^{t+\frac{N}{2}}{\left(\theta \left(k\right)-\bar{\theta }\right)}^{2}$$

In summary, the above features characterize the dynamic response of sensor attitude during compaction from multiple perspectives, including cumulative change, instantaneous rate, overall intensity, and local uniformity. Their engineering significance is that cumulative change reflects the overall compaction trend, angular velocity and RMS indices describe the intensity and energy level of the compaction process, and tilt together with local variance reveal the structural stability and uniformity of the asphalt mixture. By jointly extracting these features, a more discriminative set of input variables can be provided for subsequent regression modeling.

All features are aligned with K(t) according to the sample time index. To reduce redundancy and collinearity, features are selectively screened—depending on the model—using (i) mutual-information/Pearson-based thresholding, (ii) recursive feature elimination (RFE), and (iii) permutation importance from tree-based models.

### 3.4. Establishing the Mapping Between Smart Particle Monitoring Data and Compaction Degree

To standardize data input, let the single sampling interval be Δt; at time $t_{i}=i\Delta t$, the corresponding attitude angles and compaction degree are, respectively,

(26) $$\Theta_{i}=\left(\theta_{i},\phi_{i},\psi_{i}\right),K_{i}=K\left(t_{i}\right)$$

Align the raw compaction degree sequence to the attitude time axis via spline interpolation to obtain $\{K_{i}{\}}_{i=1}^{N}$ with matching indices. As defined in Section 3.3, compute the attitude feature vector $x_{i}$ at each time and form the sample set ${\left\{\left(x_{i},K_{i}\right)\right\}}_{i=1}^{N}$.

Under a unified data-processing pipeline, the following regressors are built in parallel on the attitude-feature sample set and compared:

1. Linear models: ridge regression and quadratic polynomial regression;
2. Tree-based methods: ExtraTrees regression;
3. Distance-based methods: KNN regression;
4. Neural networks: fully connected MLP;
5. Ensemble learning: LightGBM regression.

Hyperparameters for each regression model were systematically set based on preliminary trials and prior experience. For Ridge regression, the regularization parameter alpha was set to 1.0. Polynomial regression used a degree of 2, and the coefficients were fitted after standard scaling. K-Nearest Neighbor regression employed k = 5 with Euclidean distance. The Multi-Layer Perceptron (MLP) included two hidden layers with 64 and 32 neurons, respectively, using the ReLU activation function and the Adam solver, with early stopping enabled and a maximum of 2000 iterations. ExtraTrees regression was configured with 400 estimators, while LightGBM (when used) included 600 estimators, a learning rate of 0.05, subsample and column-subsample ratios of 0.9, and other default parameters. The selection of these hyperparameters ensured stable model convergence and sufficient predictive performance across all specimens. Detailed hyperparameter settings and configuration files are provided in the Supplementary Material.

## 4. Results and Discussion

### 4.1. High-Temperature Performance Verification of the Smart Particle

**Figure 4:**
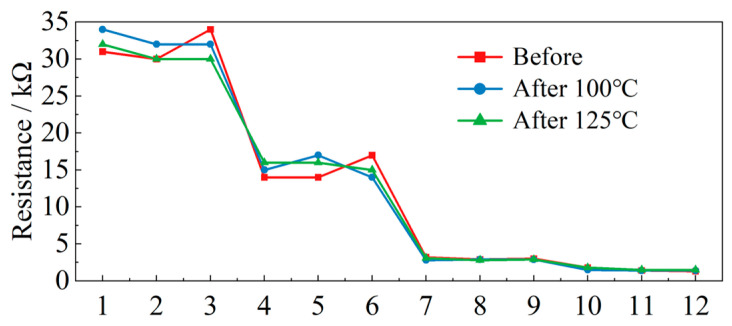
Resistance variation of the piezoresistor after isothermal holding at different temperatures: before heating, after 100 °C holding, and after 125 °C holding.

From the component-level high-temperature tests, the piezoresistor exhibits a load-dependent thermal deviation: at relatively low applied loads (≤6.11 N), the relative resistance change before and after isothermal holding is more noticeable; however, within the tested range of 0.14–6.11 N, the maximum variation is limited to a change from 34 Ω to 30 Ω. In contrast, at higher loads (≥20.30 N), the resistance–load responses before and after heating nearly coincide, with the maximum variation in the 20.30–58.09 N range being only from 1.3 Ω to 1.4 Ω. Quantitatively, the heating-induced deviation in resistance remains within 7.69–11.76% over the tested load range, which is small compared with the normal measurement uncertainty and can therefore be considered acceptable for engineering monitoring. After isothermal holding for 1 h at 100 °C and 125 °C, no obvious hysteresis, irreversible drift, or sensitivity attenuation is observed, indicating that the selected piezoresistive element maintains stable electromechanical performance under temperatures representative of asphalt mixture construction. The resistance variation of the piezoresistor after isothermal holding is presented in Figure 4.

**Figure 5:**
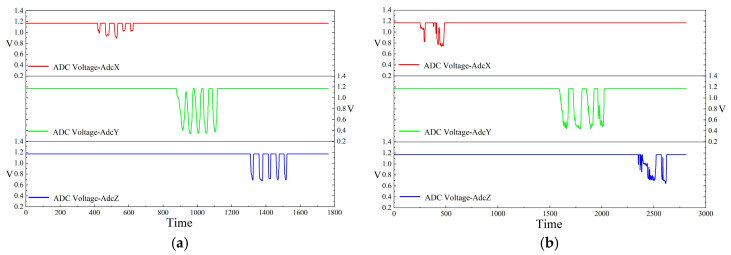
ADC voltage signals of the piezoresistive sensor in the X, Y, and Z directions during compaction: (**a**) Prior to thermal cycling; (**b**) after thermal cycling.

Further whole-particle high-temperature survivability tests confirm that, after embedding in 165 °C asphalt mixture and naturally cooling to ambient temperature, the smart particle remains fully functional. As illustrated in Figure 5, a comparison of the three-face pressing responses before and after the heating–cooling cycle shows high repeatability: the baseline variation of the force-sensing signal is controlled within 0.5%, and the press–release timing deviation is within 10 ms, which fully satisfies the 100 Hz sampling requirement. In addition, the peak-response amplitudes and the loading–unloading trajectories exhibit no discernible sensitivity loss, indicating that the temperature effect on the pressure-sensing module is negligible within the tested thermal cycle.

**Figure 6:**
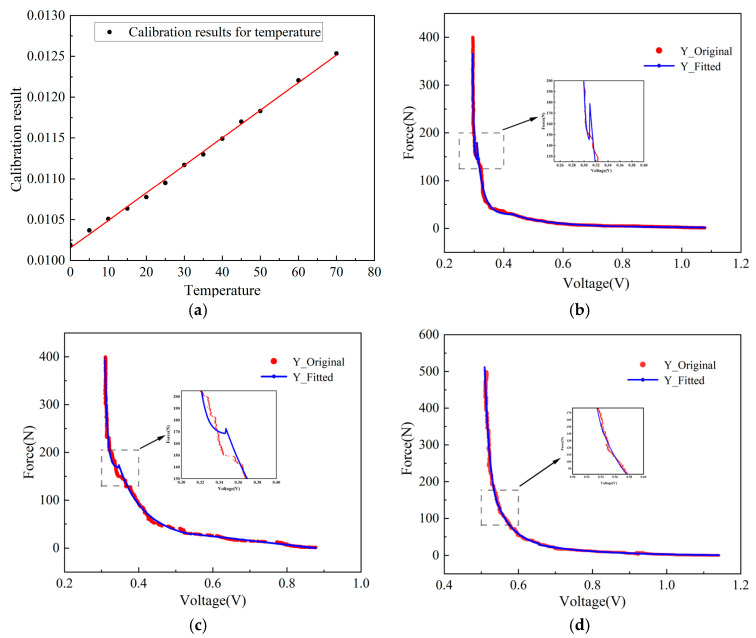
Calibration results of the temperature and force-sensing modules: (**a**) Temperature calibration curve showing the relationship between output voltage and chamber temperature; (**b**) force calibration in the X direction; (**c**) force calibration in the Y direction; (**d**) force calibration in the Z direction.

Figure 6 presents the calibration results of the temperature- and force-sensing modules. For the temperature module (Figure 6a), the output voltage exhibits a strong linear relationship with the chamber temperature over the tested range (0–75 °C), with R^2^ > 0.990. The fitted sensitivity is 0.035 mV/°C, and the maximum absolute deviation between the measured and fitted values is within 0–1.9 °C, indicating small residuals and stable sensitivity. This confirms that the temperature channel can reliably capture temperature variations during compaction and provides a trustworthy basis for mapping the internal thermal field evolution of asphalt mixture.

The stress monitoring module curve is divided into three segments, with the first two segments representing the range of lower applied forces, which corresponds to the most commonly used stress interval for monitoring. As shown in Figure 6, the power function of the curve demonstrates that, within the most relevant linear stress range (0 N to 150 N), the coefficient of determination (R^2^) consistently exceeds 0.98, indicating that the inverse linear relationship between the piezoresistor resistance and the applied load meets the accuracy requirements for precise measurements. Among the three measurement directions, the vertical channel exhibits particularly high linearity. Residuals across different loading ranges remain within reasonable bounds, demonstrating high response accuracy and stability of the force sensor in multi-directional loading conditions. Although the lateral and tangential channels show relatively larger fluctuations, their overall trends remain consistent with the applied loading pattern, confirming that the sensor effectively captures the mechanical characteristics of the compaction process.

Taken together, the calibrated outputs of the smart particle for the two key physical quantities, temperature and mechanical loading, exhibit strong repeatability and clear physical consistency with reference conditions. This performance not only provides a reliable measurement basis for subsequent vibratory-compaction monitoring but also validates the effectiveness of the sensor’s structural design and functional integration. Accordingly, the smart particle developed in this study is capable of stable operation in complex construction environments and supports further investigation into the evolution of compaction degree.

### 4.2. Analysis and Processing of Monitoring Signals During Vibratory Compaction

**Figure 7:**
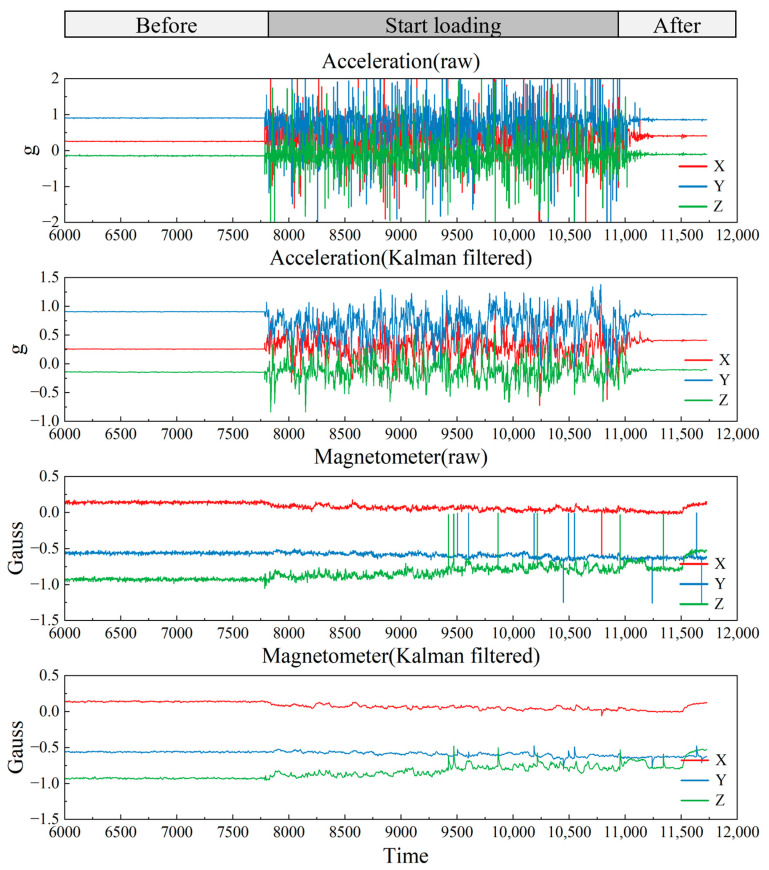
Magnetometer and accelerometer data for specimen AC ② (raw/filtered).

As the monitoring responses of different specimens exhibit similar signal-evolution characteristics during vibratory compaction, one representative case is presented here, while the results of the remaining specimens are provided in the Supplementary Material. Figure 7 shows the raw triaxial acceleration and triaxial geomagnetic signals recorded by the smart particle, together with the corresponding Kalman-filtered results. Under continuous vibratory loading, the raw signals contain evident high-frequency fluctuations and transient disturbances, which obscure the underlying variation trend and affect the subsequent attitude-angle calculation. After Kalman filtering, the random fluctuations in both the acceleration and geomagnetic signals are effectively suppressed, and the baseline trend becomes clearer, providing a more reliable input for the subsequent attitude estimation and compaction-response analysis.

**Figure 8:**
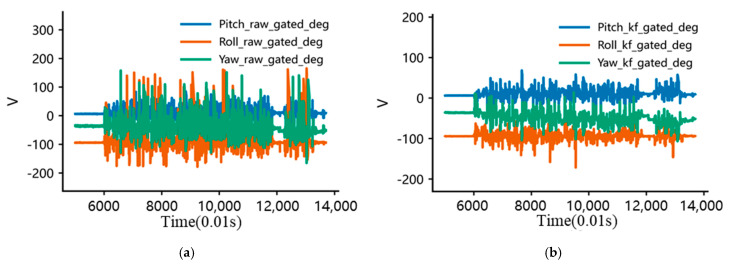
Attitude-angle estimation results of the smart particle during vibratory compaction: (**a**) Raw attitude signals obtained from the sensor measurements; (**b**) denoised attitude signals after Kalman filtering.

As illustrated in Figure 8, After Kalman filtering, the dispersion of the triaxial attitude angles converged markedly. The standard deviations of pitch, roll, and yaw decreased from 25.82°, 56.36°, and 39.89° to 13.48°, 11.14°, and 15.90°, corresponding to reductions of approximately 47.8%, 80.2%, and 60.1%, respectively. The intensity of point-to-point jumps, as quantified by the RMS of successive-sample differences, was likewise effectively suppressed: the differential RMS values of the three axes decreased from 12.91°, 22.89°, and 18.72° to 4.69°, 4.00°, and 5.01°, representing reductions of about 63.6%, 82.5%, and 73.2%, respectively. In addition, the proportion of large jumps (|Δθ| > 10°) declined from 9.78%, 8.73%, and 10.52% to 3.35%, 2.67%, and 3.46%, while the 95% step sizes contracted from approximately 28–35° to about 5–7°. Overall, the KF procedure substantially attenuated the residual random noise and abrupt outliers in the calculated attitude-angle signals, yielding smoother and more interpretable attitude-evolution curves and thereby providing a more reliable data basis for subsequent extraction of compaction-related stage features and mechanistic analysis from the posture signals.

**Figure 9:**
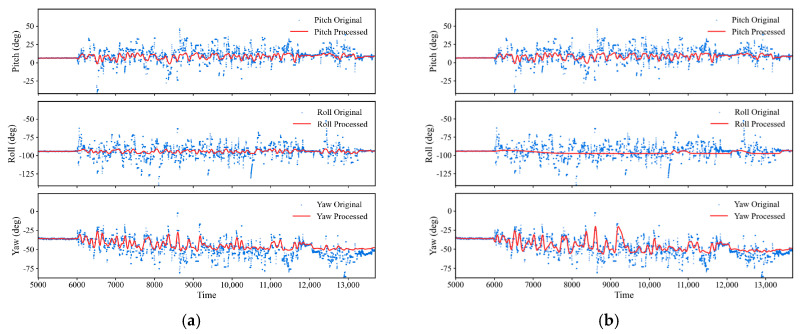

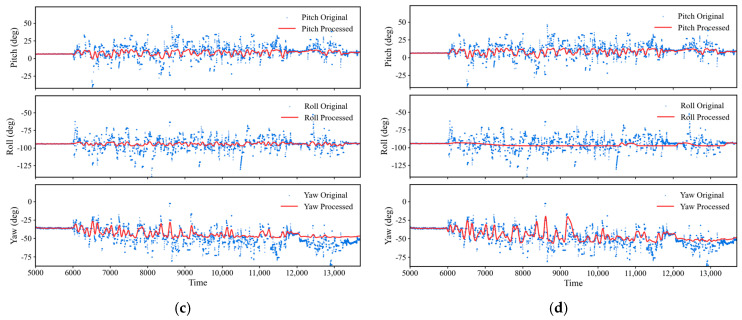
Comparison of four filtering methods: (**a**) OFF mode; (**b**) TMI mode (**c**) AWT mode; (**d**) TMI + AWT mode.

Figure 9 presents the smoothed attitude-angle data after filtering. The OFF mode obtained by ‘tanh compression + low-pass smoothing’ is shown in Figure 9a, while three additional postprocessing strategies, namely TMI, AWT, and TMI + AWT, are shown in Figure 9b–d. A comparison of the results indicates that the OFF mode already suppresses high-amplitude outliers and high-frequency fluctuations effectively in all three directions, while preserving the overall trajectory and amplitude level of the attitude sequence, thus showing good trend fidelity. Its RMSE remains relatively low (Pitch ≈ 6.37, Roll ≈ 6.54, and Yaw ≈ 6.94), indicating that the denoising treatment introduces only limited distortion to the original KF-based attitude trend. The AWT mode further suppresses a small number of residual spikes; for example, the local jump amplitude of roll, measured by P99(|Δx|), decreases from about 0.111 to about 0.102. However, the overall difference is not large, and AWT may therefore be regarded mainly as an enhanced option when residual spikes still affect interpretation. By contrast, although TMI and TMI + AWT can further reduce local jumps, with the roll P99(|Δx|) decreasing to about 0.051, they also produce more evident trend distortion, as reflected by the increase of the roll RMSE to about 7.43. Such treatments may remove short-term rearrangement responses that are physically meaningful during compaction. Therefore, these two modes were not adopted in the subsequent analysis. On this basis, AWT was selected in this study as the default denoising enhancement scheme, because it can further improve local readability when a small number of residual spikes remain, while retaining the physically meaningful posture-evolution trend.

**Figure 10:**
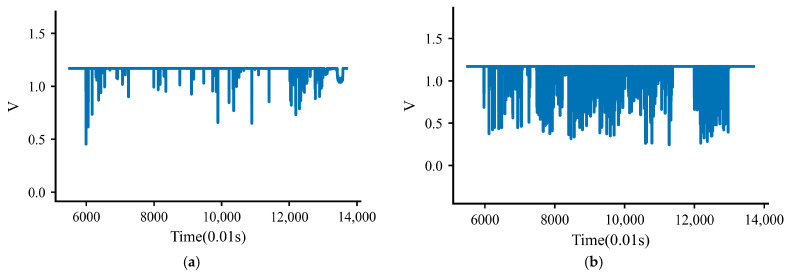

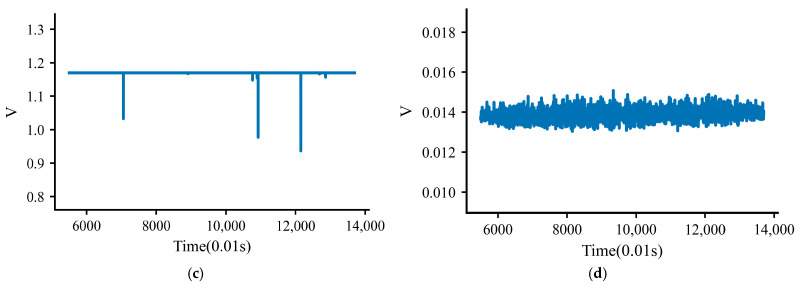
Pressure and temperature data: (**a**) X-pressure; (**b**) Y-pressure; (**c**) Z-pressure; (**d**) Temperature.

As illustrated in Figure 10, compared with the attitude signals, the pressure-channel responses exhibit greater intermittency and lower temporal stability, which is consistent with the force-transfer mechanism under vibratory compaction. Most compressive peaks occur near the instants of external impact loading, and the average time lag was less than 10 ms. The vertical channel generally shows the clearest response, indicating that the local load-transfer path is more strongly developed in the principal compaction direction. Nevertheless, the three-directional pressure curves are not smooth monotonic functions of time; instead, they fluctuate irregularly as aggregate contacts, contact surfaces, and local force chains continuously reorganize around the embedded particle. This apparent randomness should not be interpreted as measurement failure. Rather, it reflects the non-stationary and spatially heterogeneous nature of internal load transfer during densification. In contrast, the temperature signal evolves much more gradually and is mainly used to monitor the internal thermal condition of the smart particle, thereby helping confirm that the other measured signals are obtained under stable and reliable operating conditions.

**Figure 11:**
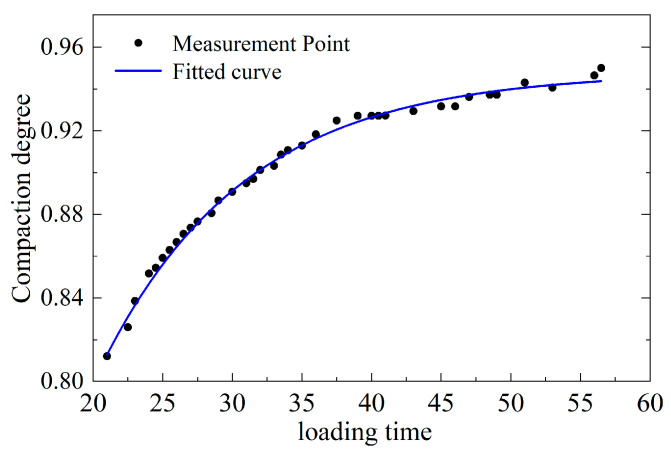
Relationship between compaction degree and loading time, including experimental measurement points and the fitted curve.

The densities obtained from the standard Marshall specimens of the six asphalt mixtures are presented in Table 3. By comparing the smart particle’s attitude outputs with the compaction degree–time curve (Figure 11), a clear two-phase evolution can be quantified. In the early stage (t = 21–37.5 s), the densification rate decreases from dK/dt ≈ 0.013 to ≈0.004 while the compaction degree continues to increase rapidly. In the late stage (t = 37.5–56.5 s), the densification rate further drops from dK/dt ≈ 0.003 to ≈0.001, and the compaction curve approaches a plateau. This two-stage reduction in dK/dt corresponds to the transition of the attitude-angle responses from fast to more gradual variation, indicating that the smart particle can delineate the rapid-to-slow densification transition and support real-time assessment of compaction quality.

Beyond consistency with compaction degree, the monitoring data offer the following advantages: Multidimensional complementarity: attitude, acceleration, magnetic, and pressure signals characterize the mixture compaction from different perspectives, creating the conditions for building multi-source fusion models. Strong field adaptability: even under high temperature, high noise, and strong disturbance, the filtered signals remain stable, demonstrating the sensor’s solid potential for engineering applications. Revealing internal mechanisms: in particular, although the randomness of the force signals reduces their direct utility, it provides experimental evidence for studying particle motion and force-chain reorganization within the asphalt mixture—insights that are difficult to obtain from conventional macroscopic tests.

### 4.3. Feature Correlation and Mechanism-Based Fitting Analysis

#### 4.3.1. Correlation Analysis

**Figure 12:**
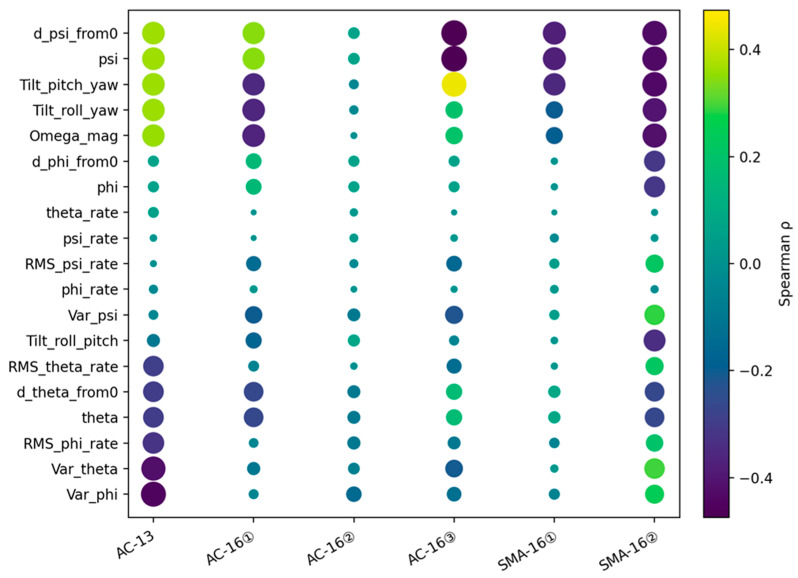
Correlation coefficient heatmap.

The Spearman correlation analysis (Figure 12) shows that the features with the most stable and strongest association with the compaction-response variable are mainly concentrated in the yaw-related group, including ψ and its relative initial offset d_ψ_from0, as well as the coupled tilt indices (Tilt_) and the composite angular-magnitude parameter Ω_mag. Specifically, d_ψ_from0/ψ exhibits moderate correlations in AC-13, AC-16①, SMA-16①, and SMA-16②, with |ρ| ≈ 0.35–0.43, while a stronger correlation is observed in AC-16③, with |ρ| ≈ 0.47. This indicates that the cumulative turning/twisting of the particle around the z-axis during vibratory compaction reflects the monotonic evolution of the compaction stage more effectively than the instantaneous attitude alone. Meanwhile, d_ψ_from0 and ψ yield identical correlation coefficients, which is consistent with the fact that they differ only by an initial constant offset and therefore preserve the same monotonic ranking in Spearman analysis. Tilt_pitch_yaw, Tilt_roll_yaw, and Ω_mag also remain in the higher correlation tier in most specimens, typically with |ρ| ≈ 0.20–0.44. Physically, these parameters describe the intensity of three-dimensional rotation, rolling, and torsional coupling of the embedded particle. 

As compaction proceeds, the aggregate skeleton becomes progressively locked and the particle mobility decreases, so the coupled posture oscillation and overall rotation intensity change monotonically and can thus be captured by the correlation analysis. It should be noted that the sign of the correlation coefficient may reverse among different specimens. This mainly results from differences in the initial placement orientation of the smart particle, which cause the Euler-angle sign convention to become mirrored among specimens. Therefore, the interpretation should focus more on the magnitude and ranking consistency of |ρ| than on the sign itself. Similarly, variance-related features such as Var_ϕ and Var_θ show relatively strong correlations in AC-13, with |ρ| ≈ 0.42–0.45, but weaker correlations in the other specimens, suggesting that the fluctuation dispersion of roll and pitch is more sensitive to certain mixture structures or installation conditions. Overall, the correlation results indicate that cumulative yaw variation (ψ/d_ψ_from0) and posture-coupling/rotation-intensity descriptors (Tilt_ and Ω_mag) are more stable and physically interpretable process features, whereas rate-, RMS-, and variance-based features are more susceptible to structural differences among specimens and the initial placement orientation of the smart particle.

The signal–compaction linkage is still conditioned by mixture-specific compaction mechanisms. Compared with the continuously graded AC mixtures, SMA mixtures tend to exhibit a more stable stone-on-stone skeleton, so the monitored response is more consistently carried by cumulative yaw-related rotation and coupled tilt features; by contrast, in AC mixtures, where compaction involves more continuous particle rearrangement and void filling across multiple size fractions, fluctuation-related features may become more sensitive under certain specimens.

**Figure 13:**
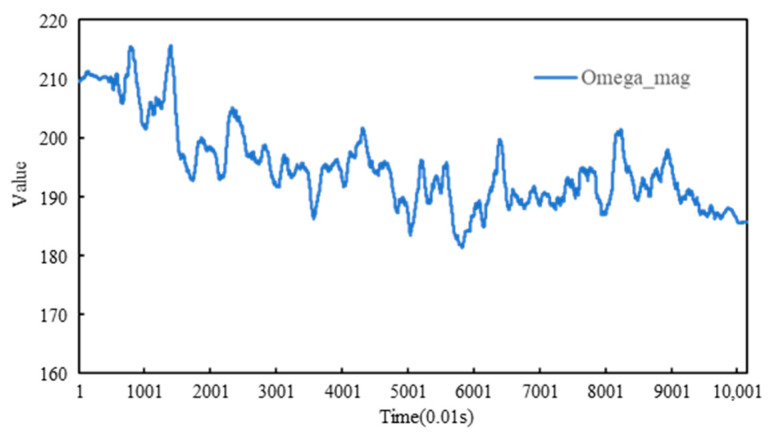
Ω_mag changing during the compaction process.

The variation curve of Ω_mag is presented in Figure 13. The composite angle magnitude Ω_mag represents the geometric norm of the triaxial attitude angles and can be interpreted as an overall “attitude intensity” indicator. Its correlation with compaction degree is strongly negative, implying continuous convergence of attitude amplitude as densification proceeds. Consistent with this interpretation, Ω_mag decreases from 209.58 in the early compaction stage to 185.63 in the late stage, with the most pronounced decline occurring within the rapid densification interval (t = 15–39 s). This behavior corroborates the mechanism that compaction suppresses high-amplitude perturbations while stabilizing low-frequency structural trends, making Ω_mag a robust feature for characterizing the transition toward a stable skeleton.

The commonality among the top five features essentially reflects a unified mechanism of constrained rotational freedom and converging lateral offsets: compaction reduces composite tilt and overall angle magnitude while driving a yaw-direction cumulative offset that changes monotonically in line with densification. This aligns with the study’s expectations for feature engineering (cumulative, intensity, and composite-tilt measures respectively represent trend, energy level, and uniformity).

#### 4.3.2. Analysis of Fitting Results Based on Compaction Mechanisms

The regression results show in Table 4 indicate significant differences in the ability of the candidate models to fit the compaction degree curves. Overall, nonlinear models, including ExtraTrees, KNN, MLP, and LightGBM, perform markedly better than the linear baselines represented by ridge regression and second-order polynomial regression. Under linear or weakly nonlinear assumptions, the model can only approximate the mapping from signal features to compaction state using a limited number of global coefficients. Once the signal contains multi-source coupling effects, systematic underfitting becomes unavoidable. This difference is also clear quantitatively: ridge regression yields R^2^ values of only about 0.170–0.380, while second-order polynomial regression reaches only about 0.260–0.600. By contrast, the nonlinear models generally achieve R^2^ values of about 0.830–0.990, which is substantially higher than the average value of approximately 0.34 for the linear models. These results indicate that the relationship between monitoring-signal features and compaction degree is not a simple additive mapping, but rather a nonlinear monotonic response jointly governed by multiple physical mechanisms. Tree-based models, nearest-neighbor regression, and neural networks can better capture feature interactions and segmented responses, and they are therefore more capable of learning the intrinsic nonlinear transition of the compaction process.

**Figure 14:**
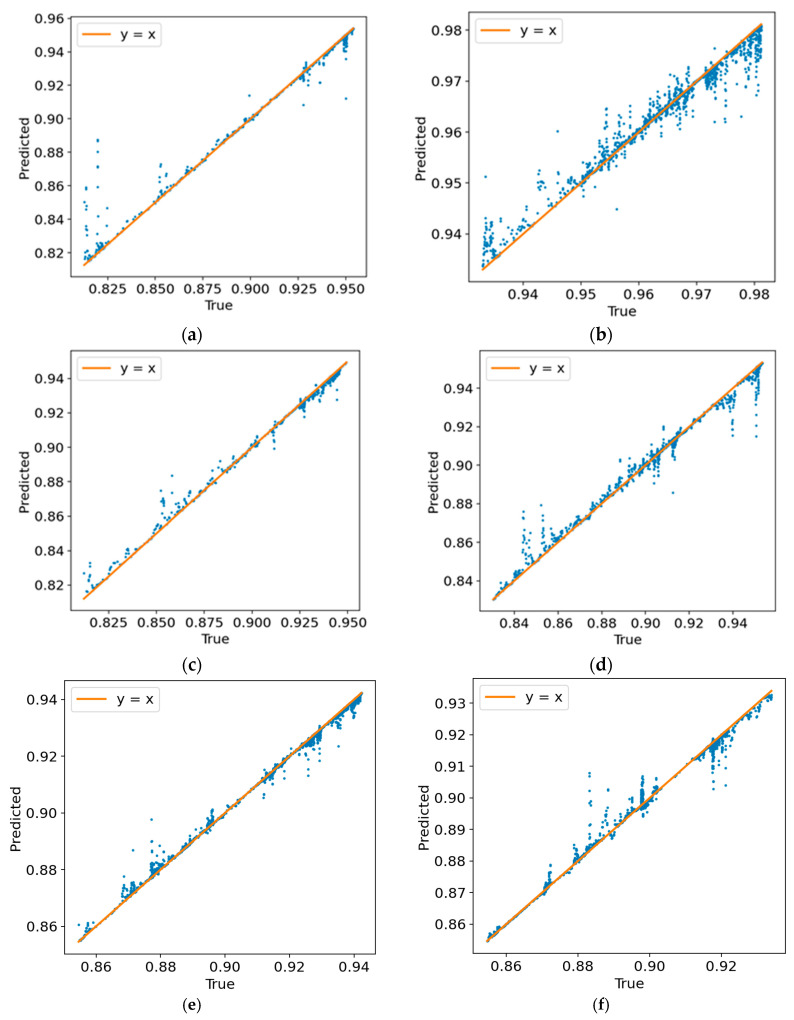
Comparison between measured and predicted compaction degrees obtained using the ExtraTrees model: (**a**) AC-13; (**b**) AC-16①; (**c**) AC-16②; (**d**) AC-16③; (**e**) SMA-16①; (**f**) SMA-16②.

From Table 4 and Figure 14, further examination of the ExtraTrees fitting results across different specimens shows that this model consistently provides highly accurate and stable predictions. The R^2^ values are generally concentrated in the range of 0.960–0.995, indicating that the model has strong capability in describing segmented nonlinear responses and feature interactions between monitoring signals and compaction state. A small number of deviations can still be observed in certain value intervals, typically in the form of locally scattered vertical bands. These deviations are mainly associated with short periods during which the signal features change rapidly while the target compaction variable responds with a slight lag. Overall, these results indicate that, after signal quality has been improved through filtering, ExtraTrees can stably reconstruct the compaction-response law reflected by the monitoring signals under different mixture and specimen conditions.

**Figure 15:**
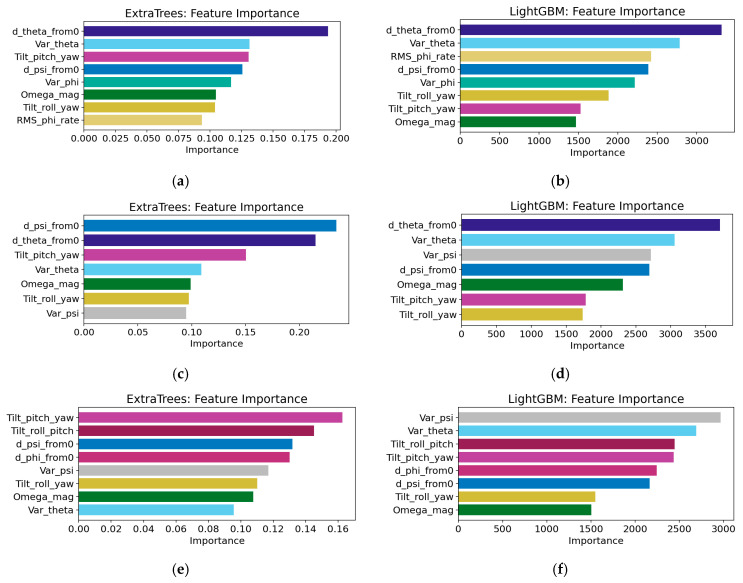
Feature-importance ranking obtained of three specimens from two machine learning models: (**a**) AC-13 from ExtraTrees model; (**b**) AC-13 from LightGBM model; (**c**) AC-16③ from ExtraTrees model; (**d**) AC-16③ from LightGBM model; (**e**) SMA-16② from ExtraTrees model; (**f**) SMA-16② from LightGBM model.

Figure 15 presents the ranking of feature importance for the ExtraTrees model and the LightGBM model on three types of specimens. The feature-importance rankings obtained from the ExtraTrees and LightGBM models show relatively clear head-feature groups across different specimens. In LightGBM, the top three features account for approximately 55–58% of the total importance, and the single most important feature contributes about 20–22%. In ExtraTrees, the importance is more concentrated in a few dominant variables, with the top three features contributing approximately 43–67%, reflecting a stronger head effect. Cross-specimen comparison further shows that cumulative posture-change features, such as d_theta_from0 and d_psi_from0, consistently rank among the top positions. In most cases, d_theta_from0 is the most important feature, whereas in some specimens, d_psi_from0 becomes dominant. Features related to posture fluctuation and coupling, such as Var_theta, Var_phi, Var_psi, Tilt_pitch_yaw, Tilt_roll_pitch, and Tilt_roll_yaw, usually occupy the remaining high ranks. Dynamic-intensity features, such as Ω_mag and RMS_phi_rate, also frequently appear among the important variables, although their exact ranking is more specimen-dependent.

From the perspective of compaction monitoring, the importance ranking indicates that the models rely mainly on two types of information that reflect the structural evolution of the particle system during compaction. The first is cumulative rotation/deflection, represented by variables such as d_theta_from0 and d_psi_from0, which describe sustained rearrangement and directional adjustment. Because these variables are more monotonic and stage-sensitive, they form stable leading contributions in tree-based and boosting models. The second is fluctuation and coupling information, represented by variables such as Var_ and Tilt_, which characterize the dispersion of posture disturbance and the intensity of triaxial coupled oscillation. These variables directly correspond to the transition from high particle mobility to restricted motion, and from frequent contact-network reconstruction to gradual structural locking. The relatively high importance of Ω_mag and RMS_phi_rate further suggests that rotational intensity and rolling rate are effective descriptors of compaction response and may be interpreted as proxies for vibration-energy input, frictional dissipation, and the transition from sliding to interlocking.

Overall, the importance results quantitatively support the physical relevance of the monitoring signals: the dominant identifiable information in the compaction process is concentrated in cumulative posture-change and posture-coupling fluctuations, whereas local differences in ranking among specimens mainly reflect differences in skeleton structure and initial boundary conditions. A further comparison between mixture types suggests that the dominant features are physically consistent within each gradation family. For SMA specimens, the repeated prominence of cumulative rotation- and tilt-related features implies that compaction is more strongly governed by the progressive stabilization of a stone-on-stone skeleton. In contrast, for AC specimens, where continuous grading allows more distributed particle rearrangement and void filling during compaction, variance- or fluctuation-related features may remain more informative in some cases. This difference does not necessarily lead to a large gap in fitting accuracy, but it does indicate that AC and SMA transmit compaction information through partially different feature channels.

From the perspective of asphalt pavement construction, these findings indicated that intelligent-particle data combined with machine learning could provide a dynamic indicator of the compaction process beyond single-point density measurements. In addition, nonlinear models were shown to be suitable for online compaction-quality evaluation and could provide real-time feedback for intelligent compaction control across different mixture types. For practical field application, the smart particles are recommended to be embedded near the bottom of the middle or lower pavement layer, where the sensed response can better reflect the internal compaction state of the structural layer. To ensure the consistency and comparability of field data, a unified and standardized coordinate system should be established for particle deployment before construction. In terms of model implementation, a practical strategy is to use the laboratory-calibrated model as the initial basis and then update it through retraining with a limited amount of site-specific field data, so as to improve adaptability and reliability under real construction conditions.

Future work will focus on further validating the proposed smart-particle-based sensing framework using a larger and more systematically controlled dataset. Since the present study is mainly intended to verify the feasibility of compaction sensing and to explore the relationship between process-response features and compaction state, the cross-specimen generalizability of the current models still requires more rigorous evaluation under expanded specimen numbers, broader mixture conditions, and more refined validation protocols. In addition, the method will be further extended toward more diverse compaction scenarios and improved engineering applicability in future studies.

## 5. Conclusions

This study developed an integrated framework for in situ monitoring and evaluation of internal responses during asphalt mixture vibratory compaction by combining intelligent-particle multi-source sensing, signal preprocessing, feature extraction, and compaction degree mapping. The experimental results demonstrate that the proposed smart particle can operate stably under the high-temperature and strong-disturbance conditions associated with asphalt mixture compaction, and that the processed posture signals can effectively characterize the staged evolution of the compaction process.

The smart particle was verified to possess adequate thermal stability, mechanical survivability, and calibration reliability for asphalt mixture monitoring. Component- and system-level tests showed that high-temperature exposure at 100 °C and 125 °C did not cause obvious hysteresis or irreversible drift, and the device remained fully functional after embedment in 165 °C hot mixture. In addition, the temperature and force channels both exhibited high calibration linearity, with *R*^2^ > 0.99 for temperature calibration and *R*^2^ > 0.98 in the most relevant force range, providing a reliable basis for subsequent monitoring and interpretation of compaction responses.

During vibratory compaction, the monitoring signals captured not only the final compaction state but also the staged evolution of internal densification. After Kalman filtering, the standard deviations of pitch, roll, and yaw decreased by about 47.8%, 80.2%, and 60.1%, respectively, while the differential RMS values were reduced by about 63.6%, 82.5%, and 73.2%, indicating that filtering markedly improved the stability and interpretability of the attitude signals under strong vibratory disturbance. The compaction curve further showed a clear two-phase densification process, with the densification rate decreasing from about 0.013 to 0.004 in the early stage and from about 0.003 to 0.001 in the late stage. Correlation analysis indicated that cumulative yaw-related features, coupled tilt indices, and the composite angular-magnitude parameter Ω_*mag*_ were the most stable and physically interpretable descriptors of compaction evolution. In particular, Ω_*mag*_ showed a strong negative correlation with compaction degree and decreased from 209.58 in the early stage to 185.63 in the late stage, confirming that compaction is accompanied by progressive suppression of large-amplitude posture disturbances and stabilization of the internal skeleton.

The regression and feature analysis results confirm that the mapping from posture-derived descriptors to compaction degree is meaningful, nonlinear, and physically informative. Compared with linear baselines, nonlinear regressors performed markedly better: second-order polynomial regression reached about 0.26–0.60, whereas the nonlinear models generally achieved *R*^2^ values of about 0.83–0.99. Among them, ExtraTrees provided the most accurate and stable predictions across the six specimens, with *R*^2^ values of about 0.961–0.995 and RMSE values of about 0.002–0.007. Feature-importance analysis further showed that cumulative posture-change features and posture-coupling/fluctuation features dominate the prediction process: in LightGBM, the top three features account for about 55–58% of the total importance, while in ExtraTrees the corresponding contribution is about 43–67%, indicating a clear head-feature effect. In addition, AC and SMA specimens exhibit partially different dominant response channels, suggesting that the proposed framework captures not only the nonlinear evolution of compaction degree but also mixture-specific compaction mechanisms. Overall, integrating intelligent-particle sensing with nonlinear learning provides a quantitative basis for online compaction-quality evaluation and supports real-time feedback for intelligent compaction control.

## Figures and Tables

**Table 1:** Physical properties of aggregates and bitumen used in the mixtures.

Aggregate Property	Value	Bitumen Property	Value
Type	Basalt	Bitumen type	#90 petroleum bitumen
Crushing value (%)	19.5	Penetration (25 °C, 0.1 mm)	83.1
Abrasion value (%)	20.3		
Flaky particle content (%)	7.3	Ductility (15 °C, cm)	>100
Apparent density (g/cm^3^)	2.733		
Water absorption of aggregate (%)	0.9	Softening point (R&B, °C)	51.2

**Table 2:** Gradation ranges of asphalt mixtures (% passing).

Sieve Size (mm)		19	16	13.2	9.5	4.75	2.36	1.18	0.6	0.3	0.15	0.075
AC-13	Lower limit	100	100	90	68	38	24	15	10	7	5	4
	Upper limit	100	100	100	85	68	50	38	28	20	15	8
	Middle value	100	100	95	76.5	53	37	26.5	19	13.5	10	6
AC-16	Lower limit	100	90	76	60	34	20	13	9	7	5	4
	Upper limit	100	100	92	80	62	48	36	26	18	14	8
	Middle value	100	95	84	70	48	34	24.5	17.5	12.5	9.5	6
SMA-16	Lower limit	100	90	65	45	20	14	14	12	10	9	8
	Upper limit	100	100	85	65	32	24	22	18	15	14	12
	Middle value	100	95	75	55	26	19	18	15	12.5	11.5	10

**Table 3:** Standard Marshall specimen density measurement results.

	AC-13	AC-16①	AC-16②	AC-16③	SMA-16①	SMA-16②
$\rho_{0}\;$ g/cm^3^	2.278	2.215	2.201	2.401	2.317	2.306

**Table 4:** Fitting performance of the various models.

Model	AC-13		AC-16①		AC-16②		AC-16③		SMA-16①		SMA-16②	
	R^2^	RMSE	R^2^	RMSE	R^2^	RMSE	R^2^	RMSE	R^2^	RMSE	R^2^	RMSE
Ridge	0.185	0.041	0.173	0.011	0.222	0.032	0.384	0.026	0.169	0.020	0.343	0.018
Polynomial (d = 2)	0.479	0.033	0.264	0.010	0.488	0.026	0.434	0.025	0.346	0.018	0.599	0.014
ExtraTrees	0.975	0.007	0.961	0.002	0.992	0.003	0.984	0.004	0.995	0.002	0.988	0.002
KNN (k = 5)	0.969	0.008	0.965	0.002	0.987	0.004	0.983	0.004	0.987	0.003	0.975	0.003
MLP (64, 32)	0.882	0.016	0.561	0.008	0.959	0.007	0.869	0.012	0.865	0.008	0.833	0.009
LightGBM	0.947	0.010	0.871	0.004	0.973	0.006	0.954	0.007	0.965	0.004	0.961	0.004

## Data Availability

The original contributions presented in this study are included in the article/Supplementary Materials. Further inquiries can be directed to the corresponding authors.

## Supplementary Materials

The following supporting information can be downloaded at https://www.mdpi.com/article/10.3390/s26061822/s1. The attachment includes: (1) Compaction degree and monitoring data of all specimens. (2) Fitting results of each model. (3) Material testing of Smart-Particle in the preliminary study. (4) Filter Parameter Information Table. (5) Photos of the test site.

## Abbreviations

The following abbreviations are used in this manuscript:

AC	Asphalt Concrete
SMA	Stone Mastic Asphalt
PAC	Porous Asphalt Concrete
CT	Computed Tomography
ICMV	Intelligent Compaction Measurement Value
GPR	Ground-Penetrating Radar
UAV	Unmanned Aerial Vehicle
ADC	Analog-to-Digital Converter
MODWT	Maximal Overlap Discrete Wavelet Transform
DWT	Discrete Wavelet Transform
RMSE	Root-Mean-Square Error
KNN	K-Nearest Neighbors
MLP	Multi-Layer Perceptron
LightGBM	Light Gradient Boosting Machine
