# Supplementary material for: Real-Time Embedded Smart-Particle Monitoring for Index-Based Evaluation of Asphalt Mixture Compaction Quality
Source: Sensors (Basel). 2026 Mar 13;26(6):1822. doi: 10.3390/s26061822 (PMC13030270; doi:10.3390/s26061822)

Supplementary figures

(1) Compaction degree and intelligent-particle monitoring data

AC①

Compaction degree versus loading time.

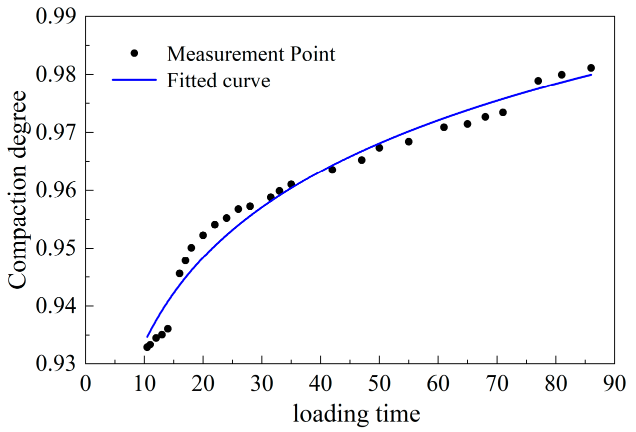

Magnetometer and accelerometer data (raw/filtered)

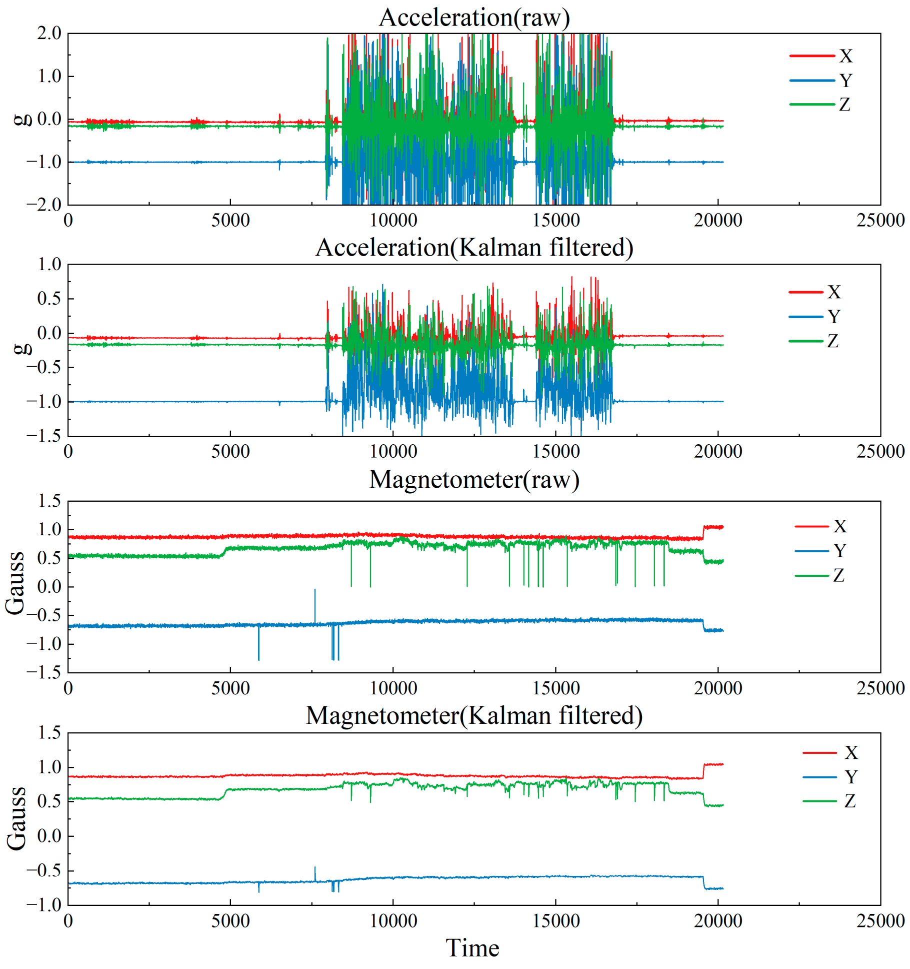

Attitude data (raw/denoised)

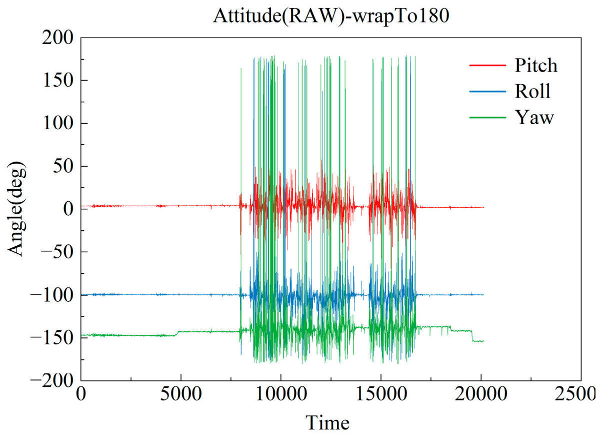

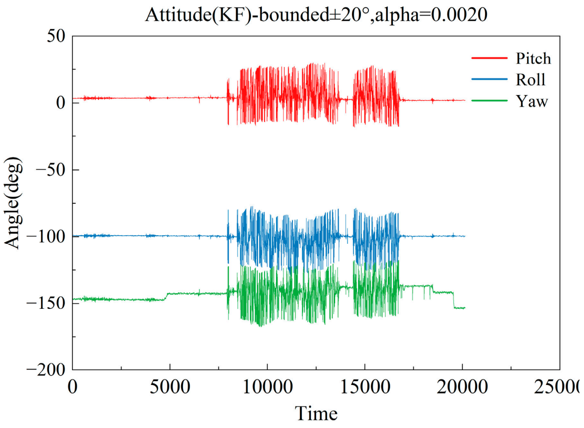

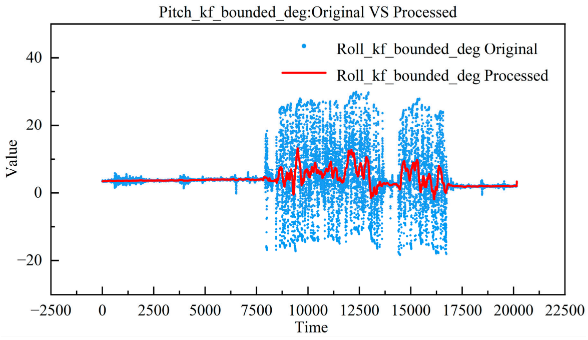

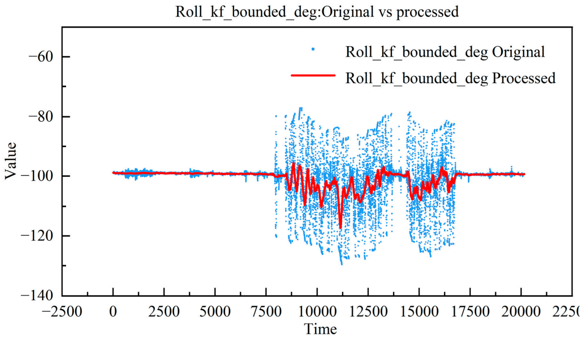

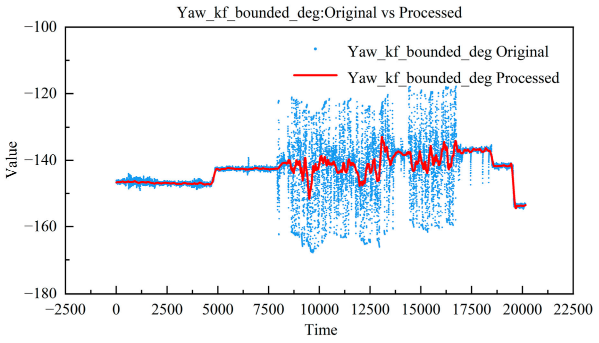

Stress and temperature data

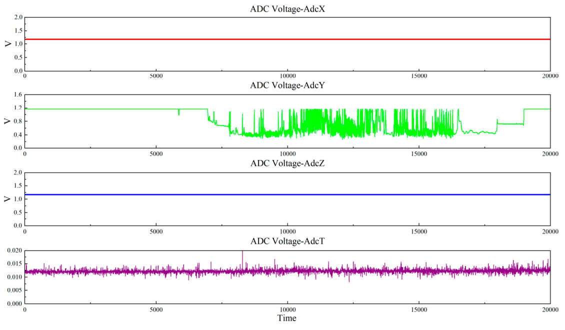

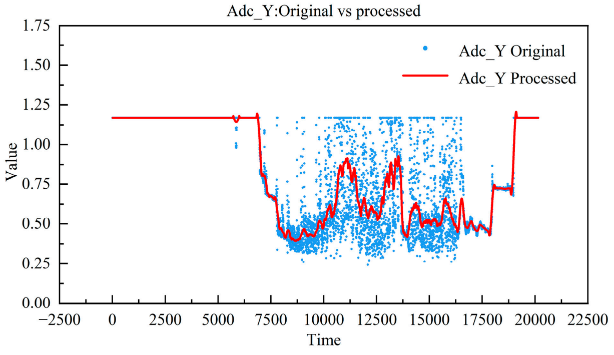

AC②

Compaction versus time

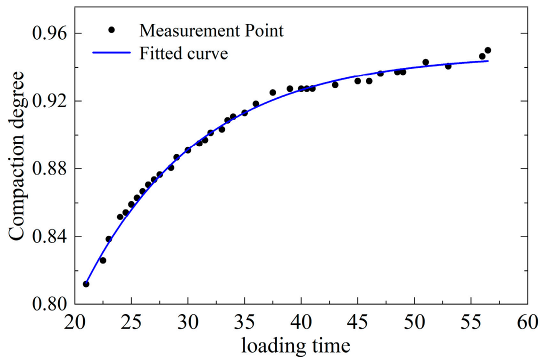

Monitoring data for AC②

Magnetometer and accelerometer data (raw/filtere)

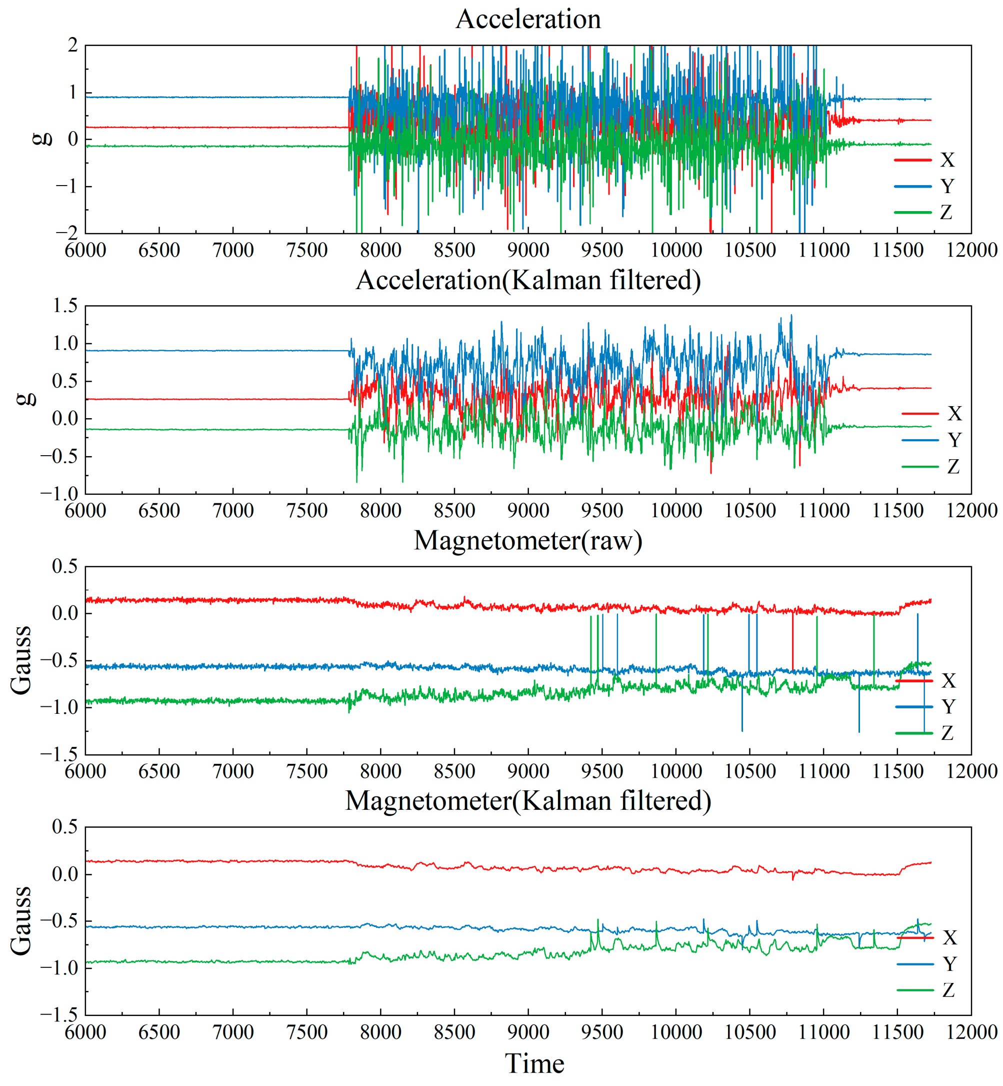

Attitude data (raw/denoised)

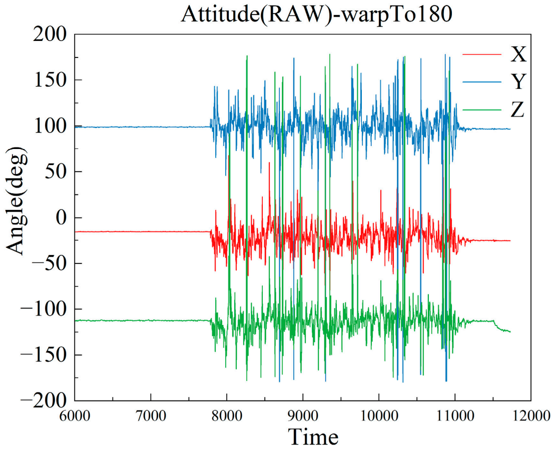

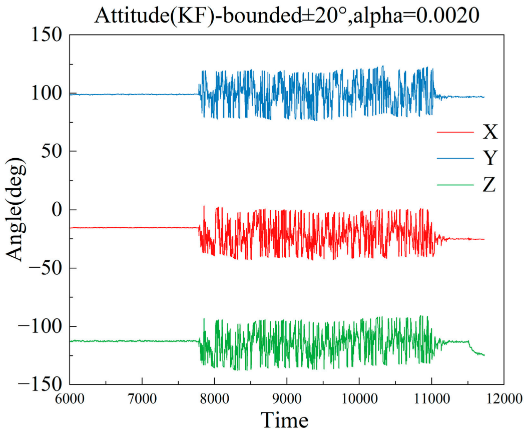

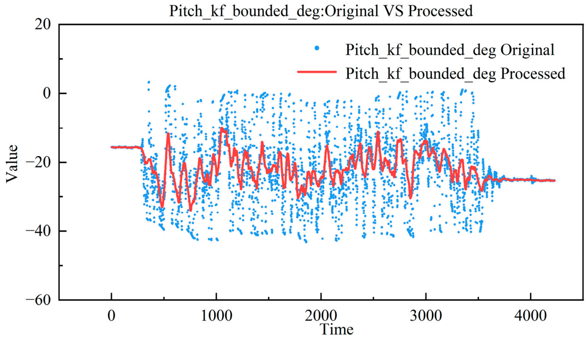

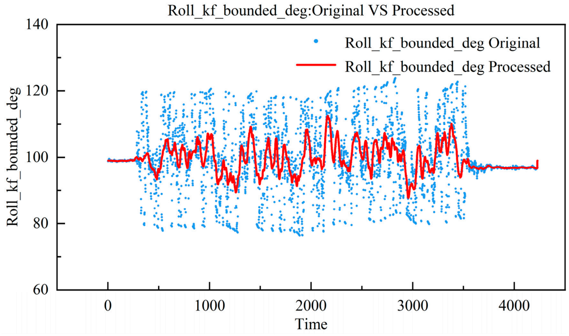

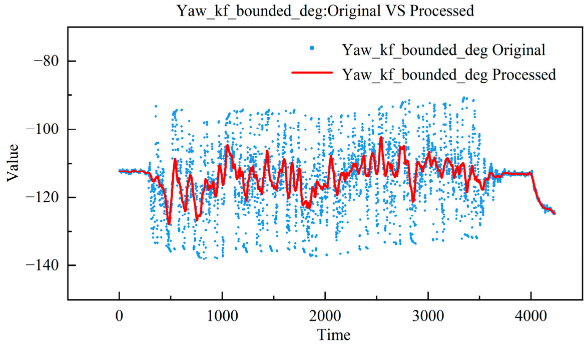

Pressure and temperature data

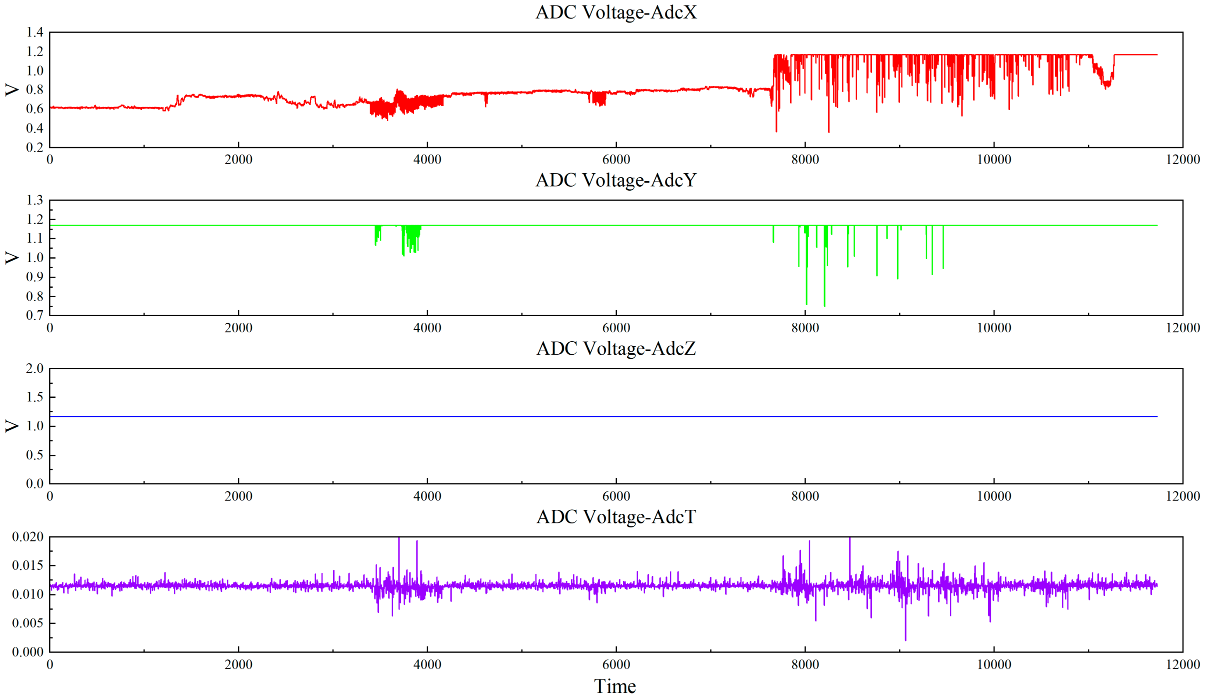

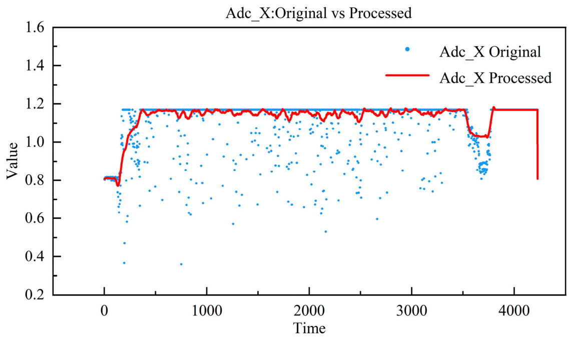

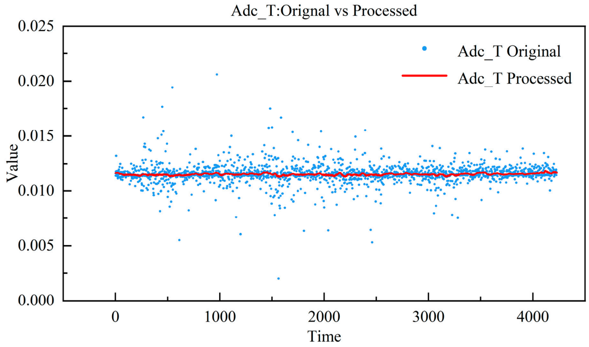

SMA①

Compaction degree versus time

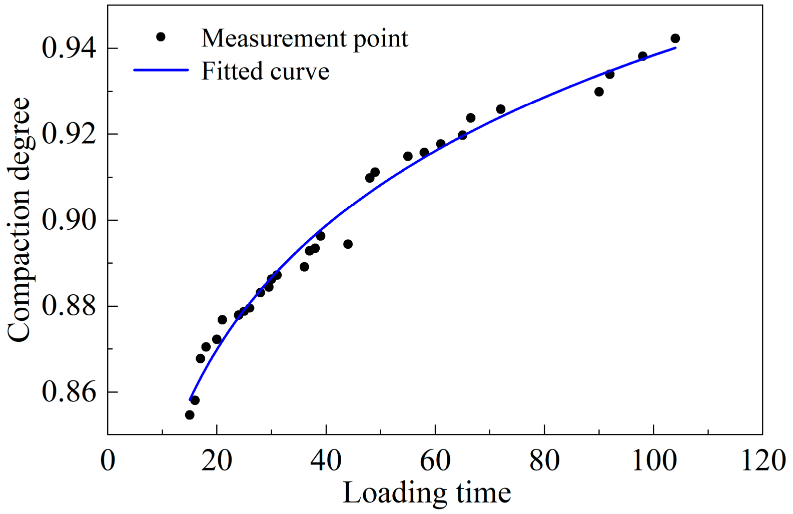

Monitoring data

Magnetometer and accelerometer data (raw/filtered)

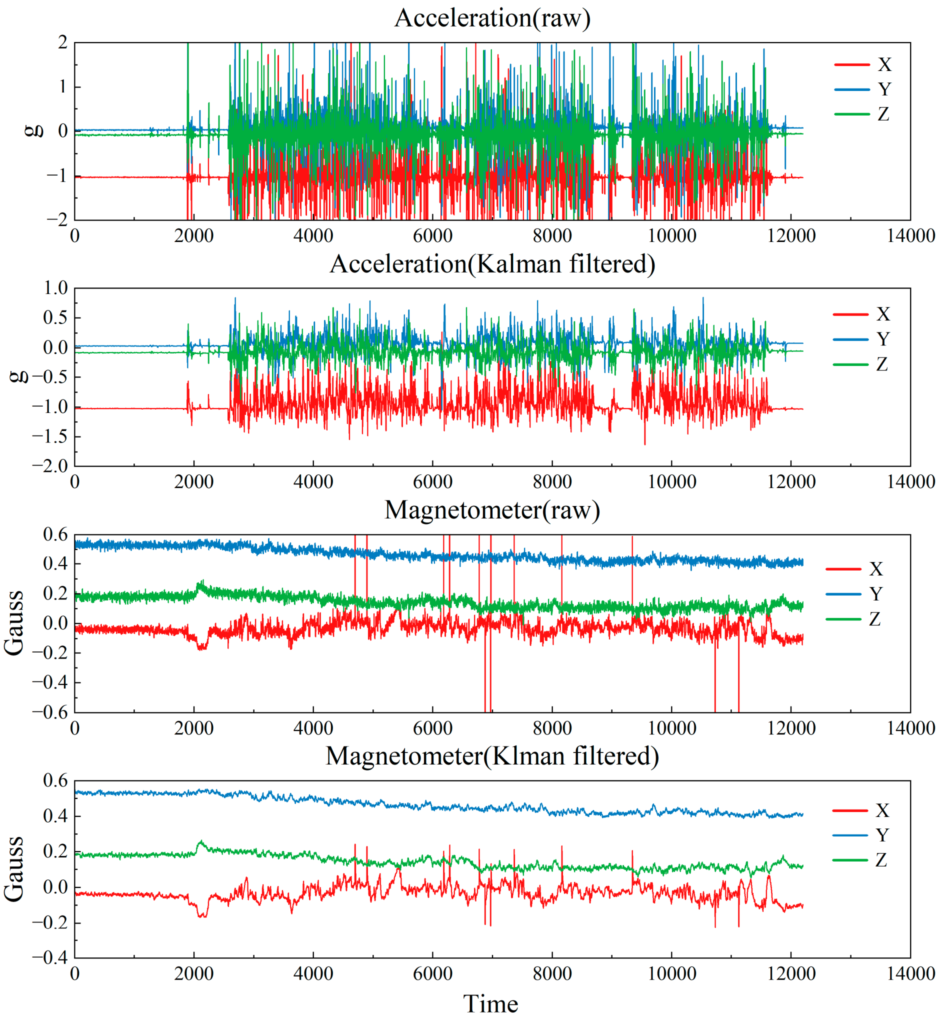

Attitude data (raw/denoised/filtered)

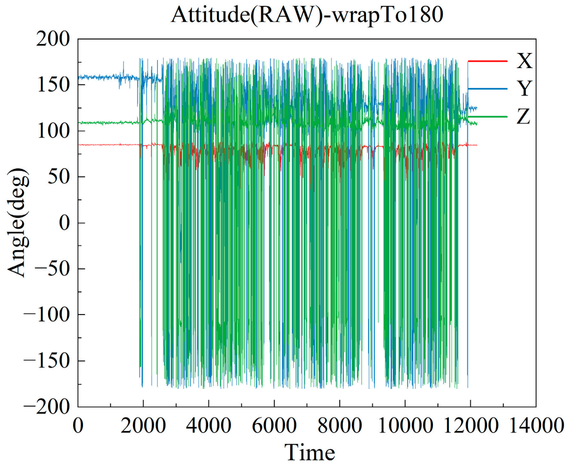

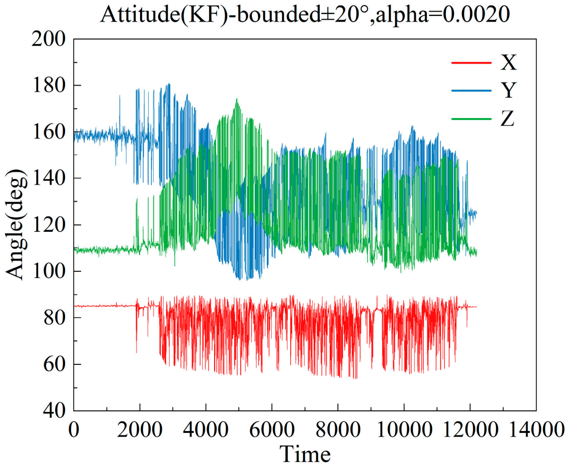

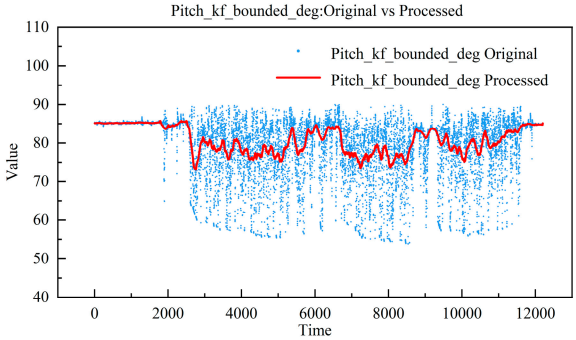

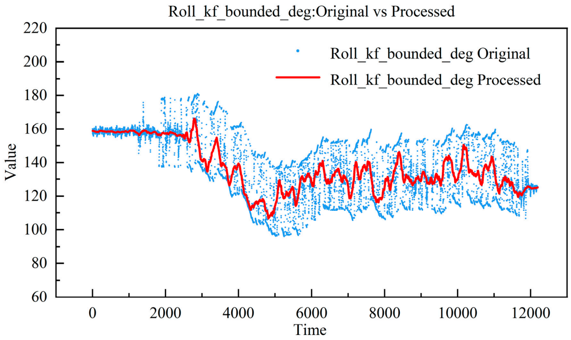

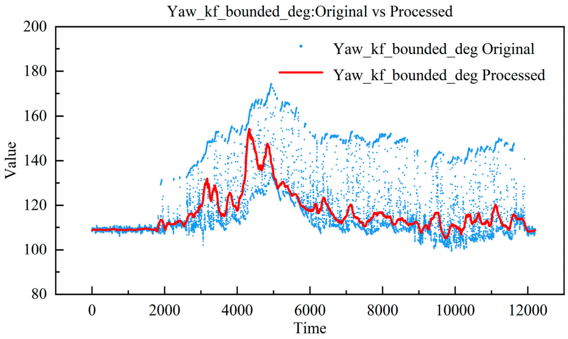

Stress and temperature data

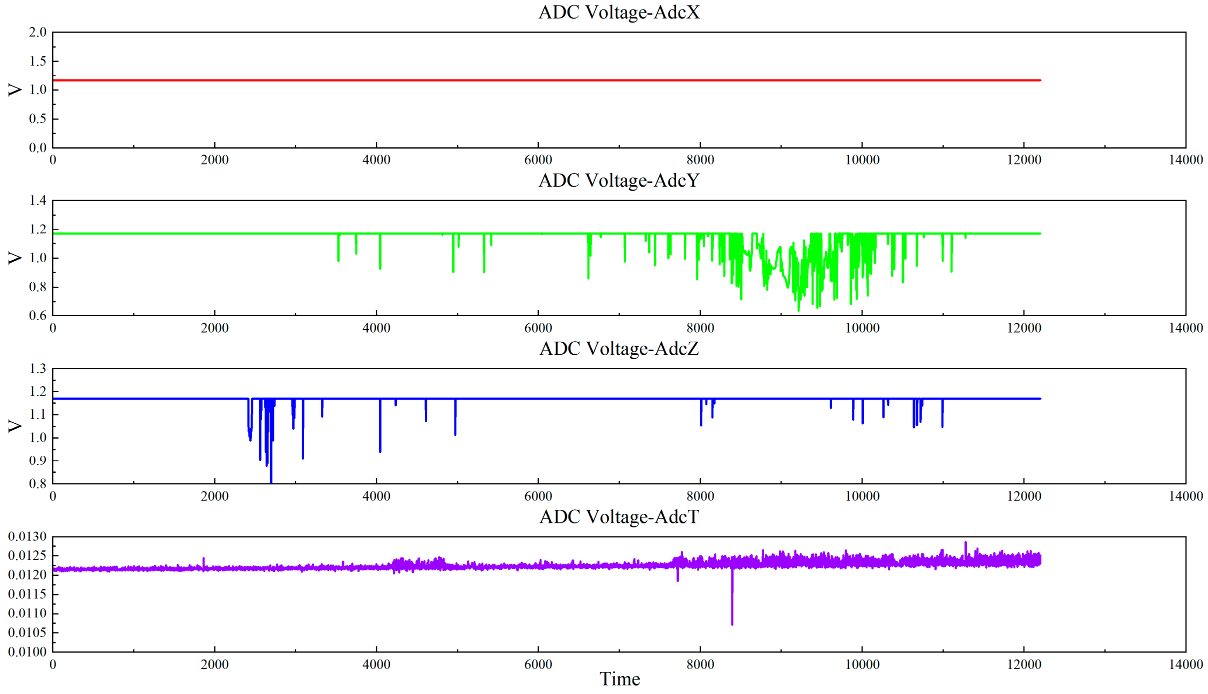

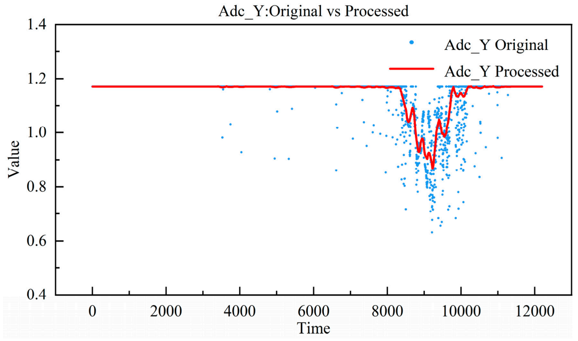

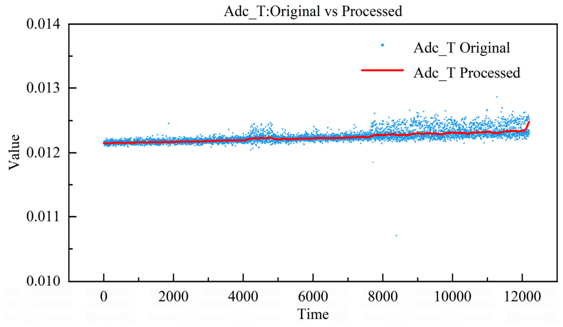

SMA② Compaction degree versus time

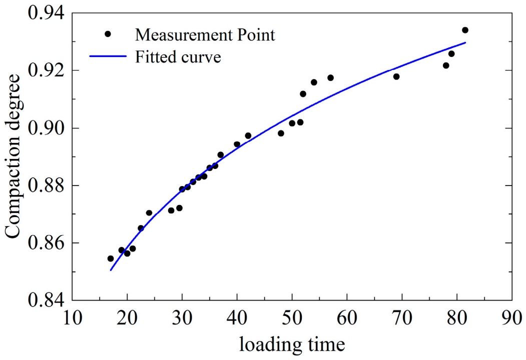

SMA② Monitoring data

Magnetometer and accelerometer data (raw/filtered)

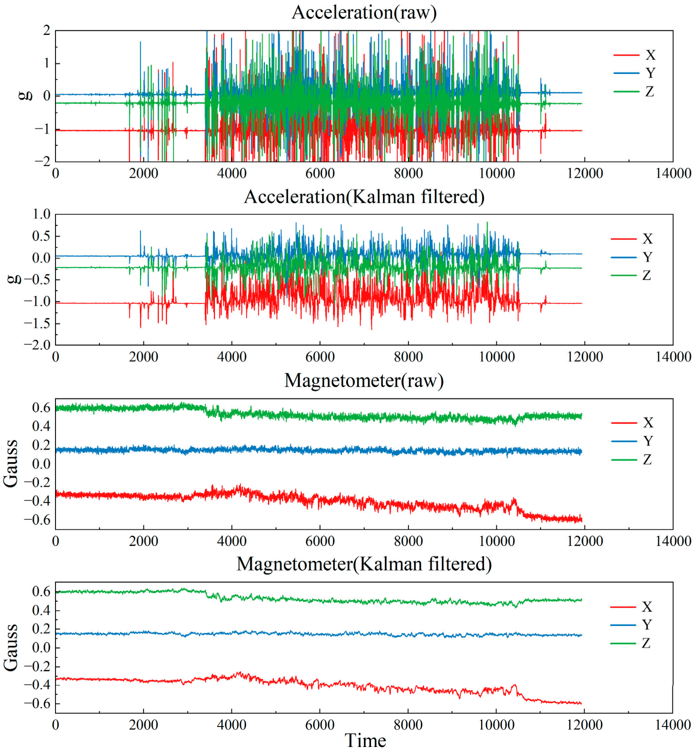

Pose data

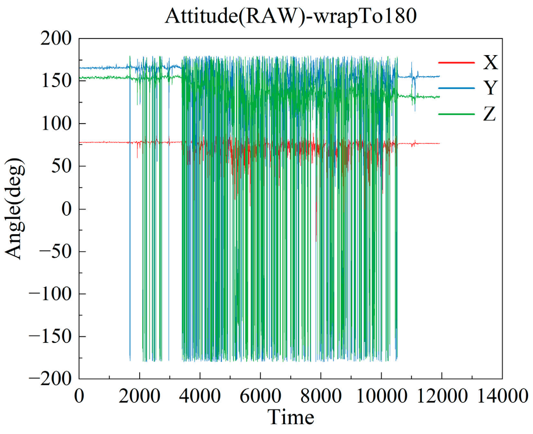

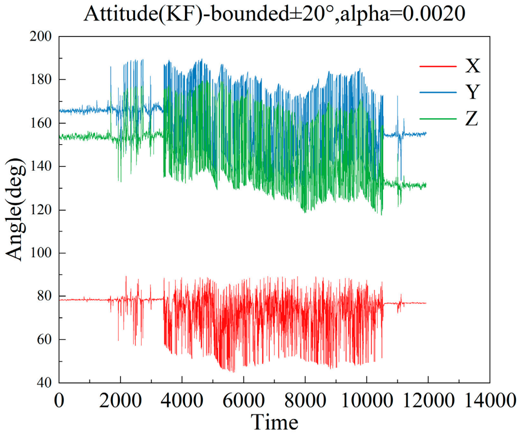

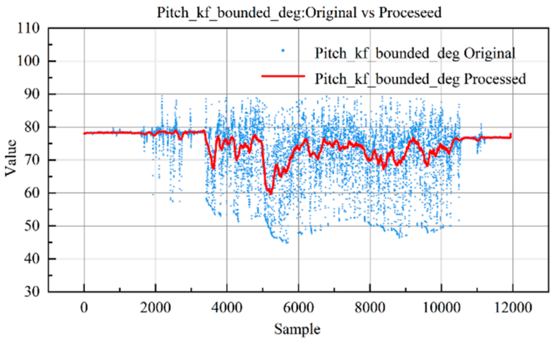

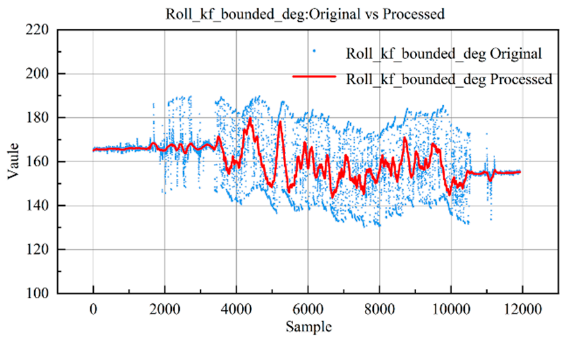

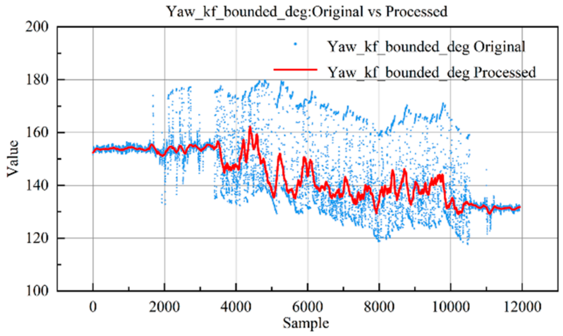

Stress data and temperature data

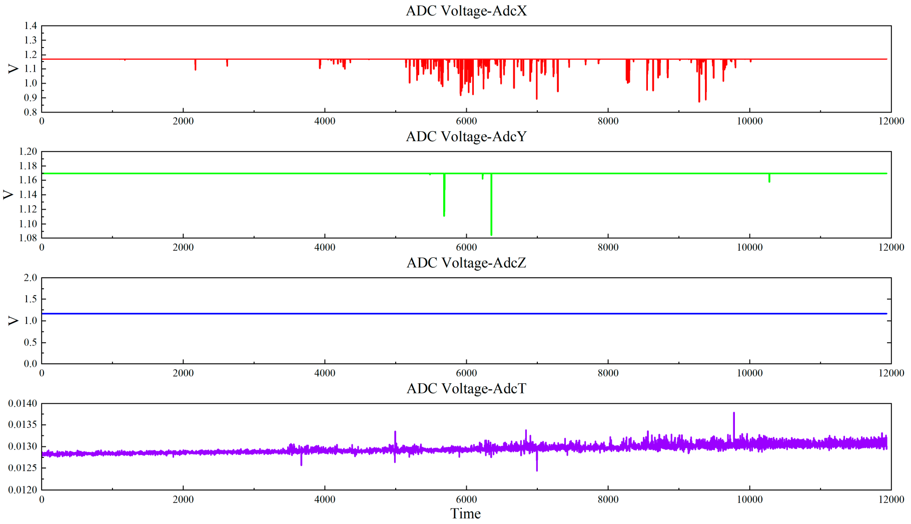

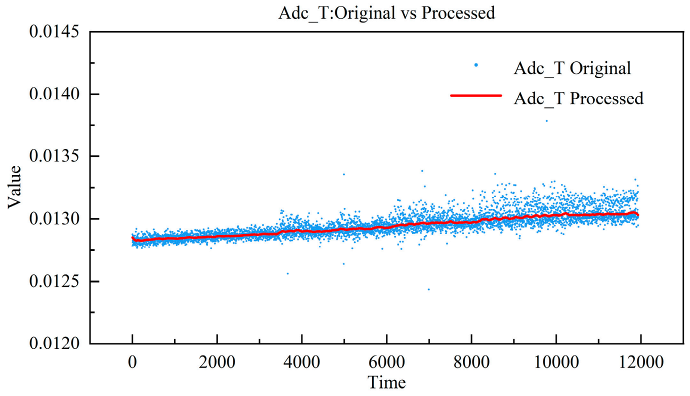

(2) Fitting results of each model:

AC①

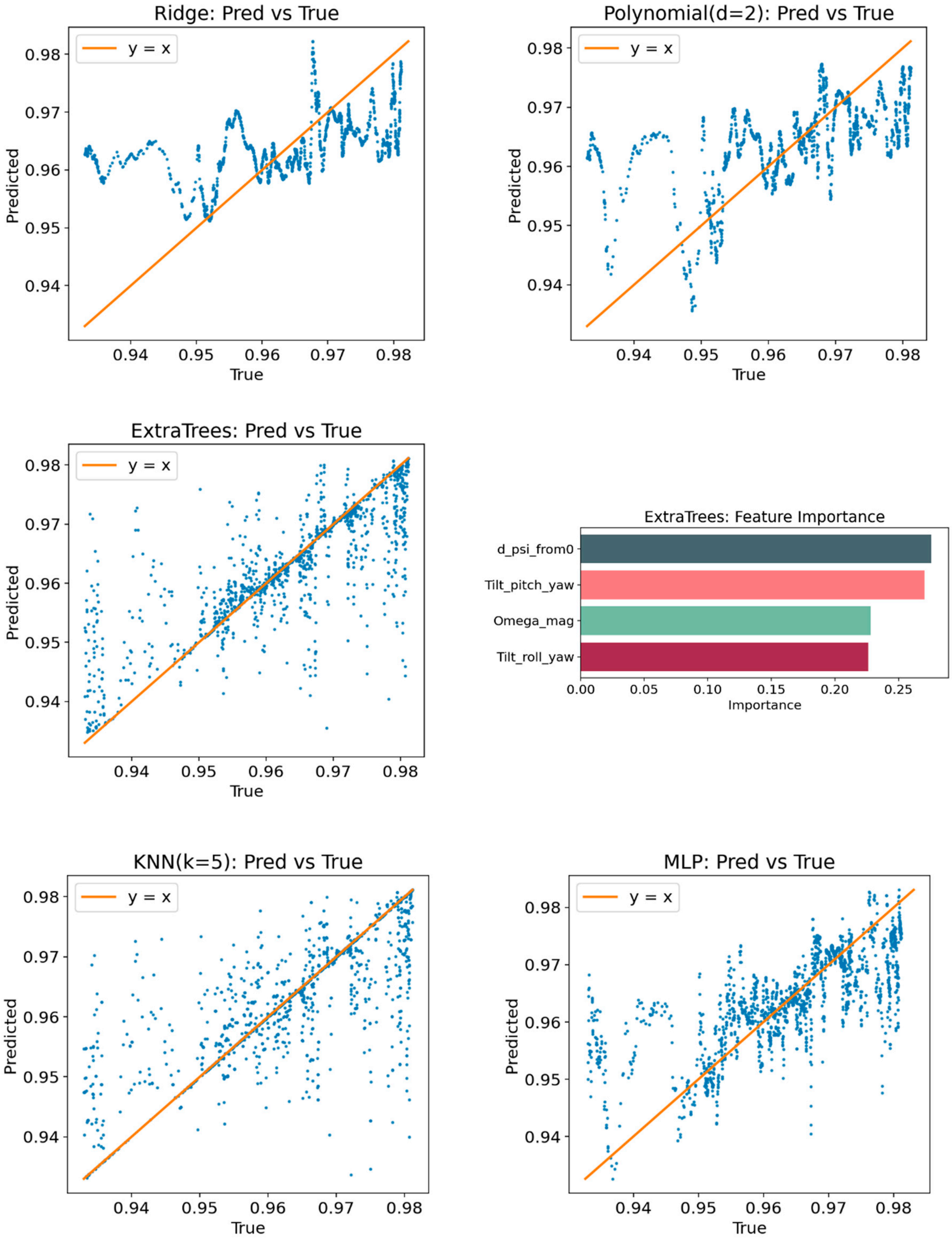

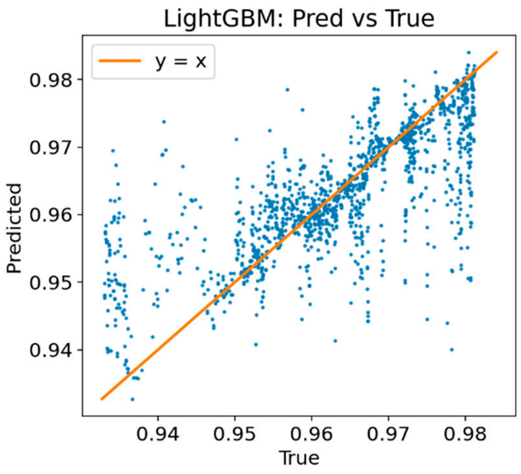
LightGBM: Pred vs True
y = x
0.98
0.97
0.96
0.95
0.94
Predicted
0.94 0.95 0.96 0.97 0.98
True

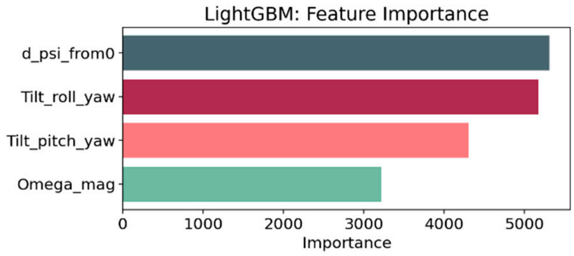
LightGBM: Feature Importance
d_psi_from0
Tilt_roll_yaw
Tilt_pitch_yaw
Omega_mag
0 1000 2000 3000 4000 5000
Importance

AC②

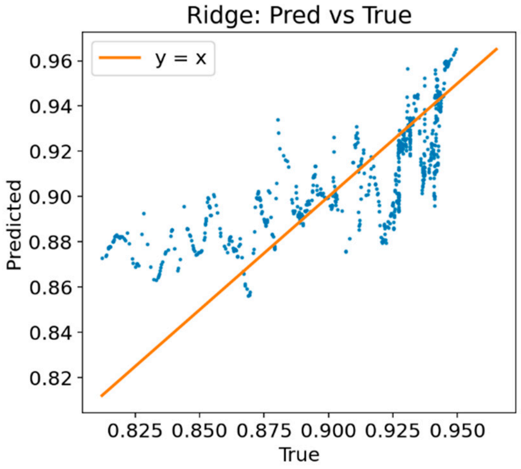
Ridge: Pred vs True
y = x
0.96
0.94
0.92
0.90
0.88
0.86
0.84
0.82
Predicted
0.825 0.850 0.875 0.900 0.925 0.950
True

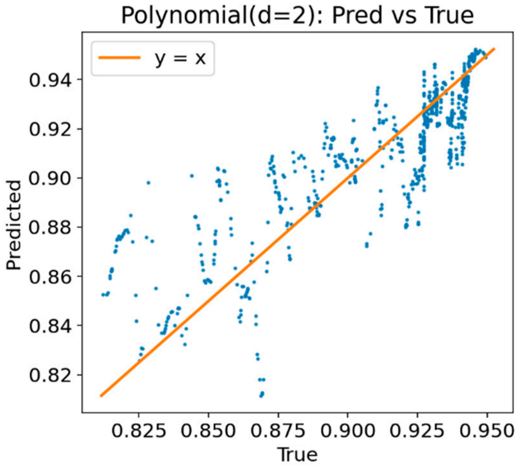
Polynomial(d=2): Pred vs True
y = x
0.94
0.92
0.90
0.88
0.86
0.84
0.82
Predicted
0.825 0.850 0.875 0.900 0.925 0.950
True

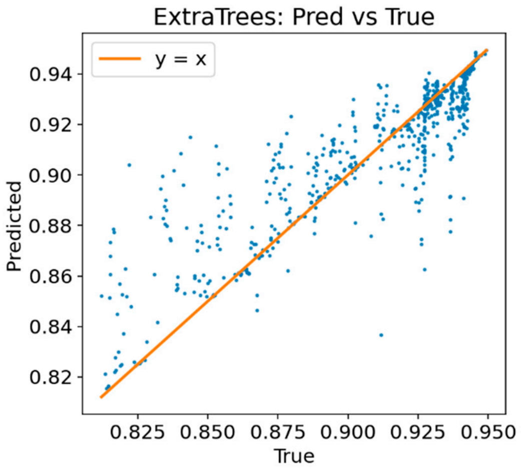
ExtraTrees: Pred vs True
y = x
0.94
0.92
0.90
0.88
0.86
0.84
0.82
Predicted
0.825 0.850 0.875 0.900 0.925 0.950
True

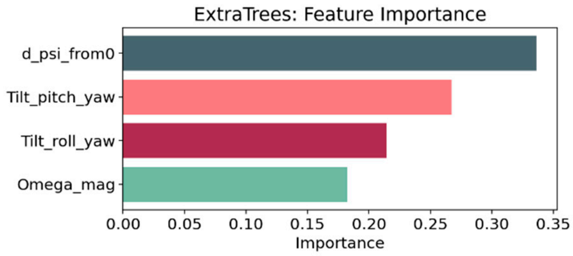
ExtraTrees: Feature Importance
d_psi_from0
Tilt_pitch_yaw
Tilt_roll_yaw
Omega_mag
0.00 0.05 0.10 0.15 0.20 0.25 0.30 0.35
Importance

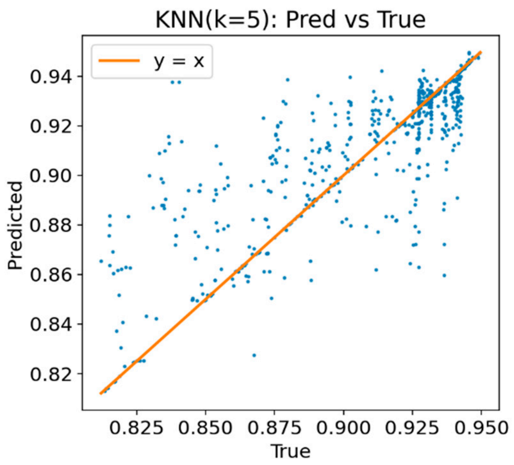
KNN(k=5): Pred vs True
y = x
Predicted
True
0.82
0.84
0.86
0.88
0.90
0.92
0.94
0.825
0.850
0.875
0.900
0.925
0.950

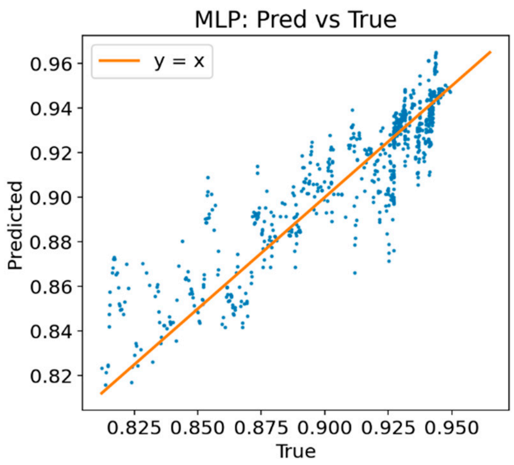
MLP: Pred vs True
y = x
Predicted
True
0.82
0.84
0.86
0.88
0.90
0.92
0.94
0.96
0.825
0.850
0.875
0.900
0.925
0.950

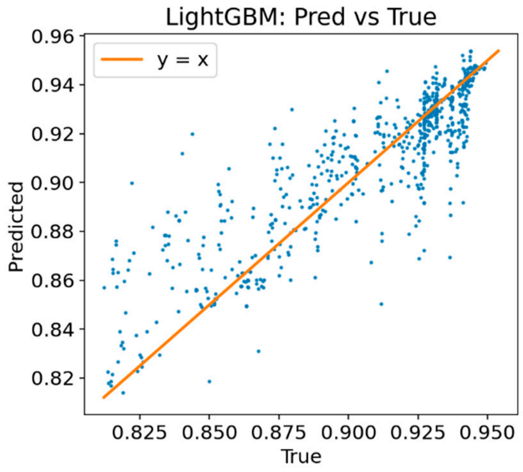
LightGBM: Pred vs True
y = x
Predicted
True
0.82
0.84
0.86
0.88
0.90
0.92
0.94
0.96
0.825
0.850
0.875
0.900
0.925
0.950

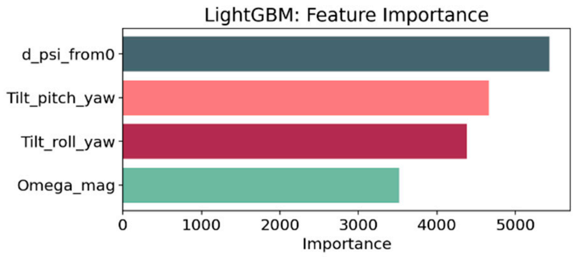
LightGBM: Feature Importance
d_psi_from0
Tilt_pitch_yaw
Tilt_roll_yaw
Omega_mag
0
1000
2000
3000
4000
5000
Importance

SMA①

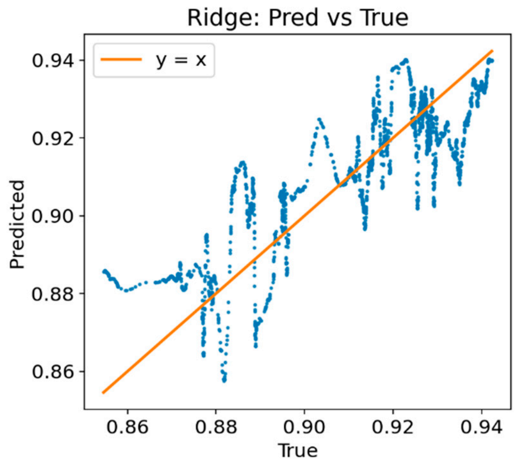
Ridge: Pred vs True
y = x
Predicted
True
0.86
0.88
0.90
0.92
0.94
0.86
0.88
0.90
0.92
0.94

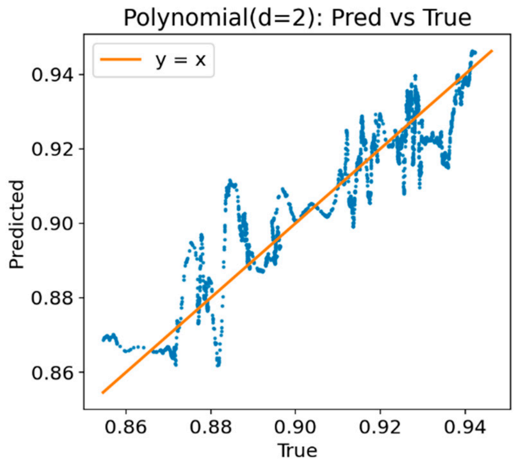
Polynomial(d=2): Pred vs True
y = x
Predicted
True
0.86
0.88
0.90
0.92
0.94
0.86
0.88
0.90
0.92
0.94

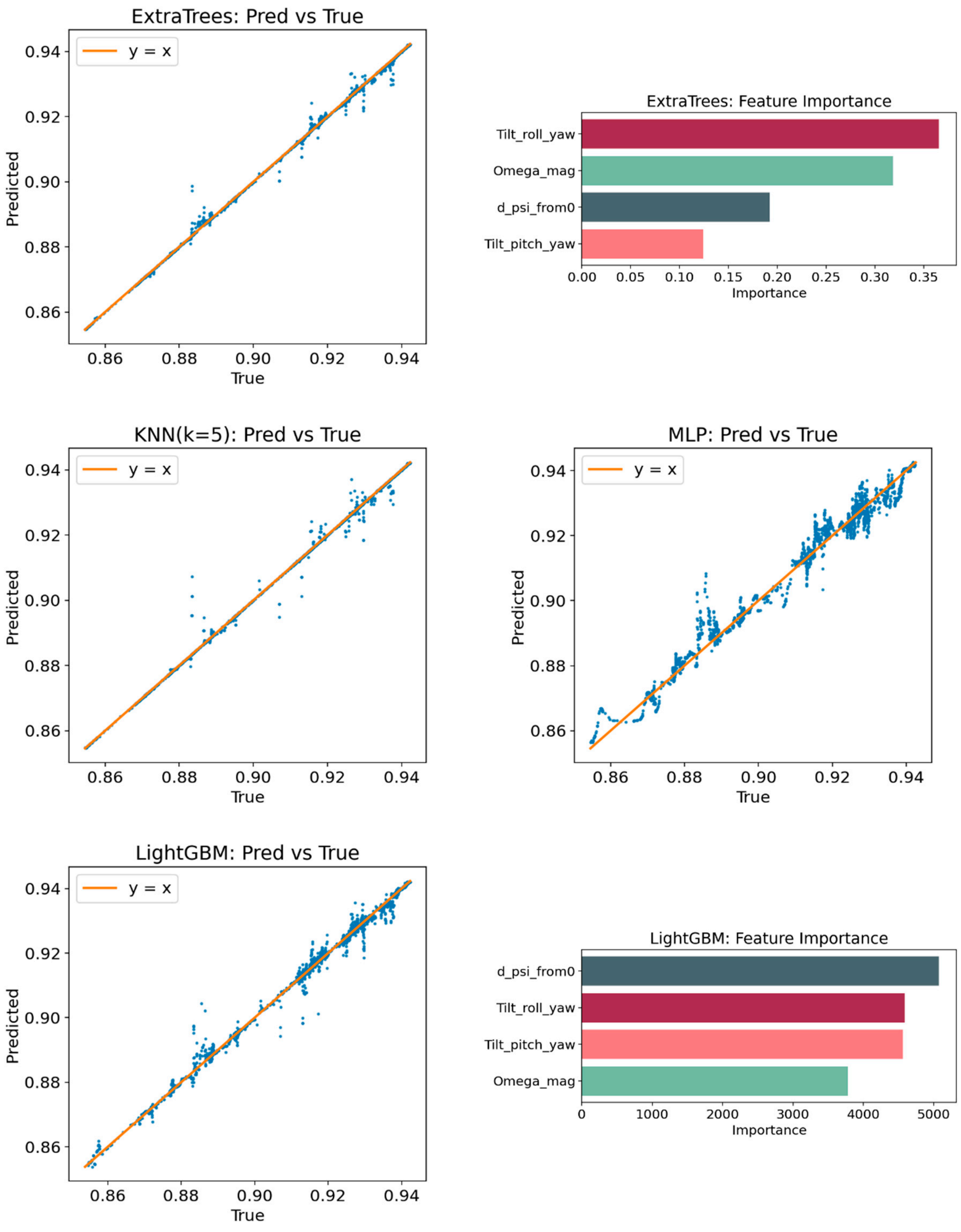
ExtraTrees: Pred vs True
y = x
Predicted
True
ExtraTrees: Feature Importance
Tilt_roll_yaw
Omega_mag
d_psi_from0
Tilt_pitch_yaw
Importance
KNN(k=5): Pred vs True
MLP: Pred vs True
LightGBM: Pred vs True
LightGBM: Feature Importance

SMA②

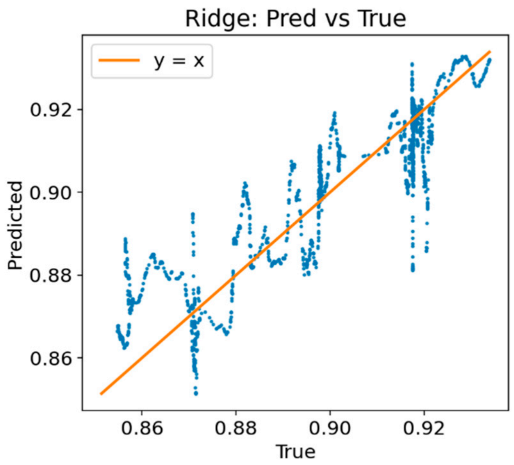
Ridge: Pred vs True
y = x
Predicted
True

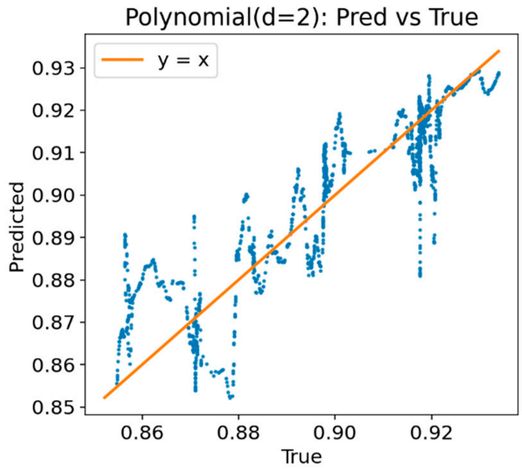
Polynomial(d=2): Pred vs True
y = x
Predicted
True

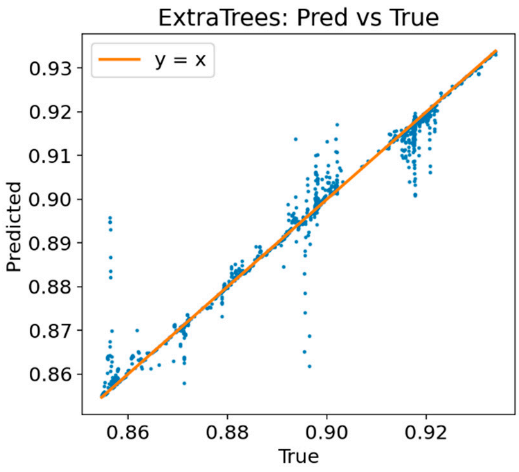
ExtraTrees: Pred vs True
y = x
Predicted
True

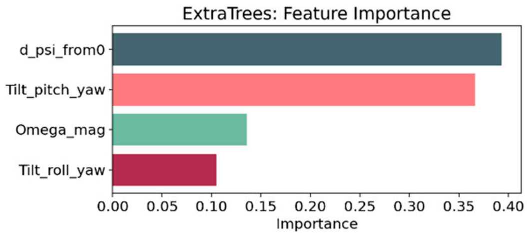
ExtraTrees: Feature Importance
d_psi_from0
Tilt_pitch_yaw
Omega_mag
Tilt_roll_yaw
Importance

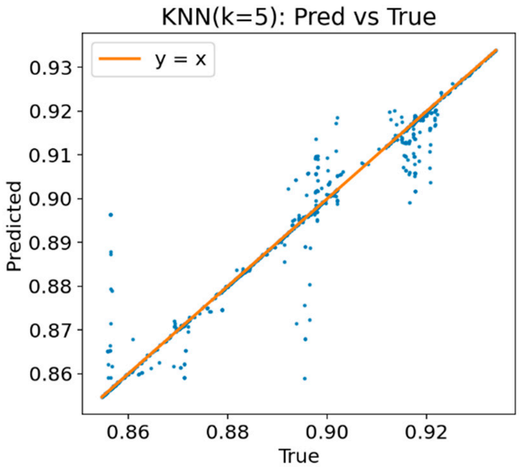
KNN(k=5): Pred vs True
y = x
Predicted
True

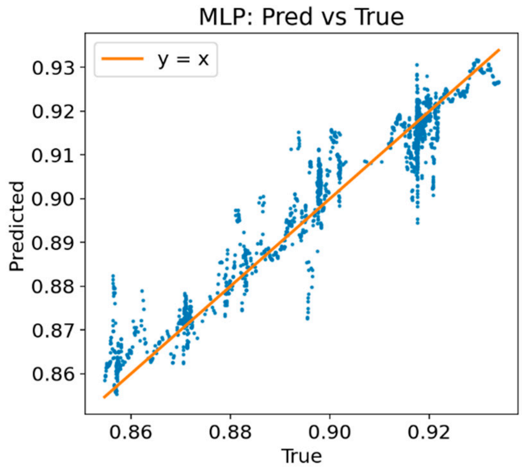
MLP: Pred vs True
y = x
Predicted
True

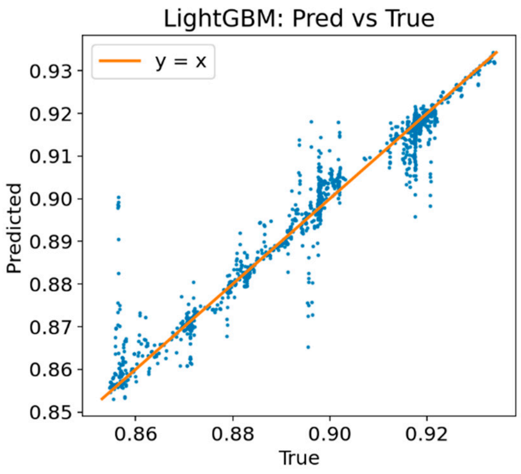

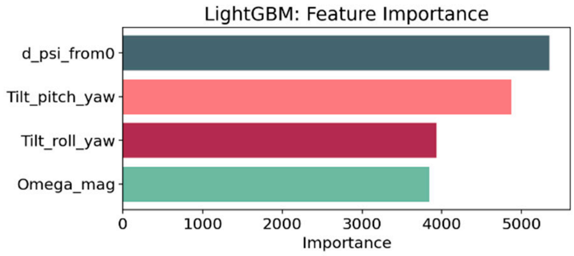

(3) Photos of the test site

Prepare for tow specimens of each mixture type

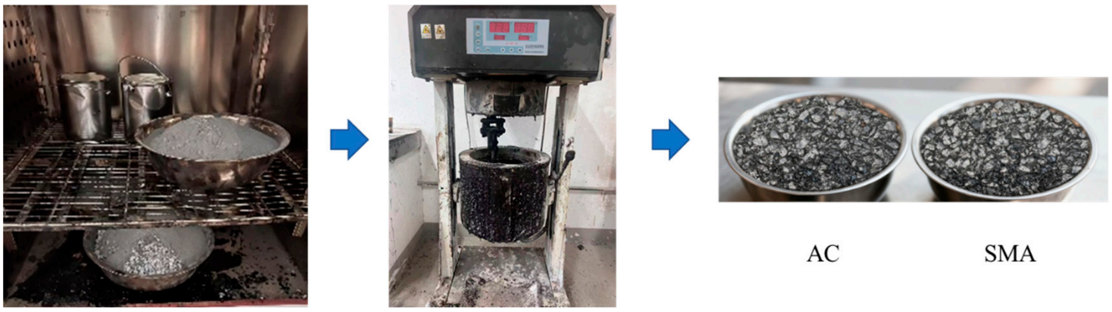

Prepare standard Marshall specimens

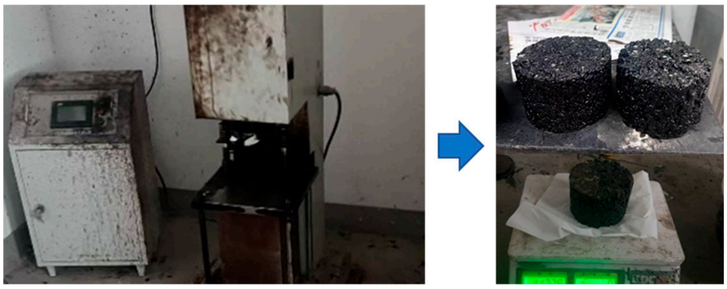

Compaction loading

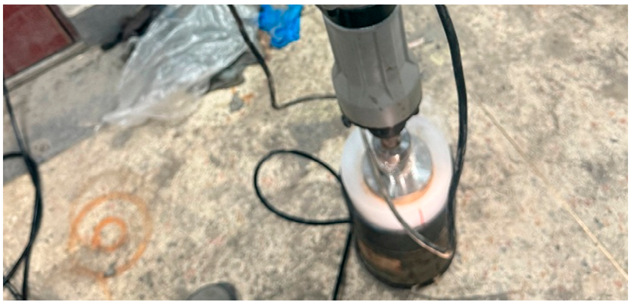

Supplement: Supplementary file 1 [file sensors-26-01822-s001.zip › sensors-4177269-supplementary.pdf]
